# CHO cell engineering via targeted integration of circular miR-21 decoy using CRISPR/RMCE hybrid system

**DOI:** 10.1007/s00253-024-13266-4

**Published:** 2024-08-09

**Authors:** Setare Adibzadeh, Shahin Amiri, Farzaneh Barkhordari, Seyed Javad Mowla, Hadi Bayat, Samaneh Ghanbari, Faezeh Faghihi, Fatemeh Davami

**Affiliations:** 1 Department of Medical Biotechnology, Biotechnology Research Center, Pasteur Institute of Iran, Tehran, Iran; 2 Student Research Committee, Pasteur Institute of Iran, Tehran, Iran; 3 Department of Molecular Genetics, Faculty of Biological Sciences, Tarbiat Modares University, Tehran, Iran; 4 Biochemical Neuroendocrinology, Institut de Recherches Cliniques de Montréal (IRCM), affiliated to the Division of Experimental Medicine, Faculty of Medicine and Health Sciences, McGill University, Montréal, H2W 1R7 Canada; 5 Cellular and Molecular Research Center, Iran University of Medical Sciences, Tehran, Iran

**Keywords:** CHO, CRISPR-Cas9, MicroRNA, Productivity, RMCE

## Abstract

**Abstract:**

Chinese hamster ovary (CHO) cells, widely acknowledged as the preferred host system for industrial recombinant protein manufacturing, play a crucial role in developing pharmaceuticals, including anticancer therapeutics. Nevertheless, mammalian cell-based biopharmaceutical production methods are still beset by cellular constraints such as limited growth and poor productivity. MicroRNA-21 (miR-21) has a major impact on a variety of malignancies, including glioblastoma multiforme (GBM). However, reduced productivity and growth rate have been linked to miR-21 overexpression in CHO cells. The current study aimed to engineer a recombinant CHO (rCHO) cell using the CRISPR-mediated precise integration into target chromosome (CRIS-PITCh) system coupled with the Bxb1 recombinase-mediated cassette exchange (RMCE) to express a circular miR-21 decoy (CM21D) with five bulged binding sites for miR-21 sponging. Implementing the ribonucleoprotein (RNP) delivery method, a landing pad was inserted into the genome utilizing the CRIS-PITCh technique. Subsequently, the CM21D cassette flanked by Bxb1 *attB* was then retargeted into the integrated landing pad using the RMCE/Bxb1 system. This strategy raised the targeting efficiency by 1.7-fold, and off-target effects were decreased. The miR-21 target genes (*Pdcd4* and *Atp11b*) noticed a significant increase in expression upon the miR-21 sponging through CM21D. Following the expression of CM21D, rCHO cells showed a substantial decrease in doubling time and a 1.3-fold increase in growth rate. Further analysis showed an increased yield of hrsACE2, a secretory recombinant protein, by 2.06-fold. Hence, we can conclude that sponging-induced inhibition of miR-21 may lead to a growth rate increase that could be linked to increased CHO cell productivity. For industrial cell lines, including CHO cells, an increase in productivity is crucial. The results of our research indicate that CM21D is an auspicious CHO engineering approach.

**Key points:**

• *CHO is an ideal host cell line for producing industrial therapeutics manufacturing, and miR-21 is downregulated in CHO cells, which produce recombinant proteins.*

• *The miR-21 target genes noticed a significant increase in expression upon the miR-21 sponging through CM21D. Additionally, sponging of miR-21 by CM21D enhanced the growth rate of CHO cells.*

• *Productivity and growth rate were increased in CHO cells expressing recombinant hrs-ACE2 protein after CM21D knocking in.*

**Graphical abstract:**

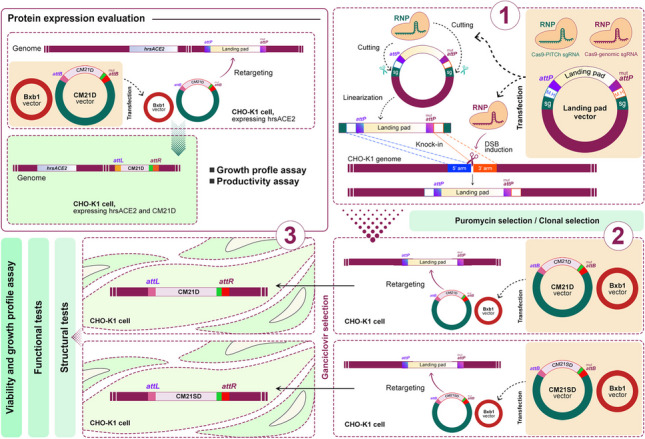

**Supplementary Information:**

The online version contains supplementary material available at 10.1007/s00253-024-13266-4.

## Introduction

Chinese hamster ovary (CHO) cells are the predominant mammalian cell line employed for the large-scale production of therapeutic proteins (Schlosshauer et al. 2022). CHO cell engineering has been applied to improve the productivity of recombinant proteins. Numerous strategies to engineer apoptosis resistance, cell proliferation, product secretion, or cell metabolism have been documented (Maccani et al. 2014; Bazaz et al. 2022; Li et al. 2022; Liu et al. 2022; Amiri et al. 2023; Xu et al. 2023; Zhang et al. 2023). One such strategy is microRNAs (miRNAs, miRs) engineering (Bayat et al. 2023; Bazaz et al. 2023; Klingler et al. 2023; Yang et al. 2023; Jari et al. 2024). miRNAs are small non-coding RNAs (ncRNAs) that play crucial roles in regulating gene expression by influencing the yield and quality of protein production due to their involvement in post-transcriptional regulation (Bayat et al. 2023). In the context of CHO cells, findings show that the expression of microRNA-21 (miR-21) has an inhibitory effect on the productivity of recombinant CHO (rCHO) cells (Jadhav et al. 2012).

On the other hand, according to cumulative reports during recent years, miRNAs have emerged as crucial players in cancers. The miR-21 has been found to be dysregulated in almost all types of cancers and plays significant roles in malignant processes of glioblastoma multiforme (GBM; grade IV) as the deadliest and most treatment-resistant primary central nervous system tumor by targeting genes involved in proliferation cell survival, invasion, and treatment resistance. Inhibiting miR-21 could be a beneficial strategy for treating glioblastoma (Larcher et al. 2019; Monfared et al. 2019; Fu et al. 2021) and may enhance the productivity of rCHO cells. Circular RNAs (circRNAs), a category of non-coding RNAs (ncRNAs), have a significant role in controlling gene expression and can act as a competitive binding site for gene regulators like miRNAs. By sponging miRs, synthetic circRNA decoys (sponges) can function as either promoters or inhibitors of tumors in cancer cells, underlining their potential importance in cancer biology and therapeutic approaches (Muller et al. 2020; Bayat et al. 2023).

Traditional development of rCHO cells relies on random integration, which can lead to expression instability and clonal variation due to the position effect (Lee et al. 2015; Kheirandish et al. 2023). To overcome these challenges, targeted integration methods have been developed. One of these methods is recombinase-mediated cassette exchange (RMCE). RMCE utilizes site-specific recombinases such as Bxb1 to facilitate the incorporation of a transgene at a pre-designated locus within the genome. This locus is expected to harbor a pre-existing cassette, which serves as the “landing pad” containing recombinase recognition sites. This method allows for more controlled and predictable gene expression (Gaidukov et al. 2018; Low et al. 2022). More recently, targeted integration strategies such as the CRISPR-Cas9 system have been developed and profoundly altered genome editing technology. This approach introduces a double-strand break (DSB) into the genome at a specific location, directed by a single guide RNA (sgRNA). Following a break in the DNA strand, repair mechanisms like non-homologous end-joining (NHEJ) or homology-directed repair (HDR) are activated at the site of damage. Gene knock-out or knock-in can result from the completion of this repair process, respectively. The pre-existing landing pad required for the RMCE/Bxb1 system can be introduced using the CRISPR/Cas9 system, providing precise control over the integration site (Gaidukov et al. 2018; Rahmani et al. 2023). A modified strategy for gene knock-in termed the Precise Integration into Target Chromosome (PITCh) system was introduced by Nakade et al. (2014), which utilizes the CRISPR/Cas9 system (referred to as CRIS-PITCh), leverages the microhomology-mediated end joining (MMEJ) repair mechanism.

Unlike HDR, MMEJ employs very short microhomologies (≤ 40 base pairs (bp)) to integrate the gene of interest precisely. The relatively short microhomology arms, which can be conveniently added via PCR or oligonucleotide annealing, prevent the need for long-range PCR for knock-in confirmation. This accelerates the on-target verification process, enhancing the system’s efficiency. Moreover, the PITCh system utilizes the in vivo linearization of the donor vector, which recognizes and cuts the PITCH sgRNA cut sites designed on the PITCh donor vector. This enables backbone-free integration (Rahmani et al. 2023). A hybrid approach that combines the precision of the CRISPR/Cas9 system with the robustness of RMCE has been employed for cell line development (Ghanbari et al. 2023). However, it is essential to emphasize that even with its outstanding accuracy, the CRISPR/Cas9 system has flaws. One of the primary issues with the CRISPR/Cas9 toolbox is the incidence of off-target effects or undesired mutations at genomic regions other than the intended target. Such off-target effects arise when the guide RNA attaches to regions of the target DNA other than the sequence for which it was designed (Atkins et al. 2021). Moreover, the CRISPR/Cas9 system’s efficiency might occasionally be low, which poses a serious obstacle to its usefulness (Rasul et al. 2022). One way to mitigate off-target effects and improve the CRISPR/Cas9 system’s efficiency involves modifying the delivery method. In particular, this can be accomplished by utilizing ribonucleoprotein (RNP), which comprises the Cas9 protein complexed with in vitro-synthesized sgRNA. The RNP complex ensures the immediate availability of a functional nuclease, which leads to rapid gene editing (“fast-on”) and enhances efficiency. The short-lived nature of the editing activity (“fast-off”) signifies that the RNP complexes are quickly degraded and removed from the cell, reducing potential off-target mutations and enhancing the precision of the gene editing process (Rasul et al. 2022; Han et al. 2023). Also, the utilization of RNP complexes in the production of recombinant proteins offers several advantages. RNPs, due to their transient activity in cells, significantly reduce the risk of unwanted genomic integration and limit the amount of residual DNA. Moreover, the precise control over gene editing offered by RNPs and their lower immunogenicity, compared to other delivery methods, further enhances their suitability (Campbell et al. 2019).

In the present experiment, we aimed to engineer an rCHO cell line capable of producing a circular miR-21 decoy (CM21D). The CM21D was designed with five bulged binding sites specific for miR-21, and its generation was facilitated through a tRNA-splicing mechanism (Bayat et al. 2023). We employed the CRIS-PITCh/RMCE hybrid system, which was recognized for its efficacy in the rapid, precise, and reliable development of transgene-expressing CHO cell lines. CRIS-PITCh was used to achieve knock-in of the Bxb1 landing pad (PITCh donor plasmid), which harbors the Bxb1 *attP* sites (required for Bxb1 system), into the upstream region of the *S100A* gene cluster using short homology arms of 30 bp. We used the RNP delivery system to avoid off-target effects and increase efficiency. The CM21D cassette, flanked by Bxb1 *attB*, was then retargeted into the integrated landing pad through the RMCE/Bxb1 system. Once retargeting the CM21D cassette into the *S100A* gene cluster, we observed a significant reduction in the doubling time of rCHO cells, an essential feature for industrial cell lines, including CHO cells. To further investigate the impact of CM21D expression on recombinant protein production, the CM21D cassette exchange was also applied to the CHO-K1 clonal cell, which co-expresses human recombinant soluble angiotensin-converting enzyme 2 (hrsACE2) as secretory recombinant proteins and pre-existing landing pad. To our knowledge, this constitutes the inaugural application of CM21D in rCHO cells as a producer cell line to augment the growth rate and productivity.

## Material and methods

### Design of sgRNAs and construction of vectors

The design of the PITCh sgRNA was based on the approach proposed by Sakuma et al. (2016), and the genome-targeting sgRNA was developed as part of our prior research (Ghanbari et al. 2023). The construction of the PITCh donor plasmid carrying landing pad construct (PITCh sgRNA cut sites-30 bp microhomology arms-*attP*-PGK-*TK*-2A-*PuroR*-SV40 polyA-mut *attP*-30 bp microhomology arms-PITCh sgRNA cut sites) and the all-in-one plasmid (pU6-PITCh sgRNA-pU6-genome-targeting sgRNA-CMV enhancer-chicken β-actin promoter-*Cas9*-T2A-*mCherry*) were also accomplished in our previous work (Ghanbari et al. 2023). To construct the circular RNA (circRNA) decoys, specifically CM21D and the circular miR-21 scrambled decoy (CM21SD), sequences were synthesized by GeneScript (Nanjing, China). Such decoys include five bulged binding sites, specifically 5 × 5′-TCAACATCAGAACATAAGCTA-3′ for CM21D and 5 × 5′- TAATGCGACCACTAAATAACA-3′ for CM21SD. These sequences were designed and predicted by the MXfold2 online tool (Sato et al. 2021) and placed under the control of a U6 promoter, which includes a 27-nt overhang necessary for inducing a higher level of tRNA expression and a *Drosophila melanogaster* polyA sequence. Subsequently, for the construction of our RMCE donor plasmids, CM21D and CM21SD sequences were subcloned upstream of the *EGFP* expression unit into the *Eco*RI and *Not*I restriction sites (*attB*-CM21D-CMV-*EGFP*-SV40 polyA-mut *attB*) and (*attB*-CM21SD-CMV-*EGFP*-SV40 polyA-mut *attB*) of a RMCE donor plasmid developed in our previous study (*attB*-CMV-*EGFP*-SV40 polyA-mut *attB*) (Ghanbari et al. 2023).

### In vitro* transcription (IVT)*

The target oligonucleotides, utilized for synthesizing PITCh and genome-targeting sgRNAs, were designed using the GeneArt™ Precision sgRNA Synthesis Kit (Thermo Fisher, Invitrogen™, Waltham, MA, USA). The oligonucleotides were synthesized by GeneScript (Nanjing, China). The subsequent steps of IVT and cleanup were carried out according to the GeneArt™ Precision sgRNA Synthesis Kit protocol.

### Cell culture and transfection to generate platform and rCHO-K1 cells

CHO-K1 cells, obtained from the Cell Bank of the Pasteur Institute in Iran, were cultured in DMEM/F12 medium fed with 10% fetal bovine serum (Gibco, Grand Island, NE, USA). The cells were incubated at 37°C in a cell incubator with 5% CO_2_ and passaged into fresh media every 3 days. A day prior to transfection, CHO cells were seeded at a density of 6 × 10^4^ cells/well in 24-well plates. For the generation of the CHO-K1 cell platform with an RNP-based delivery system, the PITCh donor plasmid (500 ng/µl) was utilized. Transfection was executed in accordance with the manufacturer’s protocol using the Lipofection 3000 reagent (Thermo Fisher, Invitrogen™, Waltham, MA, USA). After 4 h, the culture media was replaced. The first RNP complex was formed by combining the PITCh sgRNA with the Cas9 nuclease (Avan Bio Research, Karaj, Iran) at a 1:1 molar ratio, facilitating the in vivo linearization of the PITCh donor plasmid. Simultaneously, the second RNP complex was generated by assembling the genome-targeting sgRNA with the Cas9 nuclease at a 1:1 molar ratio, thereby inducing a double-strand break in the genomic DNA. The RNPs were assembled and co-transfected using Lipofectamine™ CRISPRMAX™ Transfection Reagent (Thermo Fisher, Invitrogen™, Waltham, MA, USA) according to the manufacturer’s protocol. In the context of the plasmid-mediated delivery system, cells underwent co-transfection with the PITCh donor plasmid and the all-in-one plasmid, both at a concentration of 500 ng/µl using the Lipofection 3000 reagent (Thermo Fisher, Invitrogen™, Waltham, MA, USA), according to manufacturer’s protocol. At 72 h post-transfection, antibiotic selection pressure was applied to the cells with the addition of 10 μg/mL of puromycin (Biobasic, Markham, Canada). These cells had been seeded to 6-well plates at a 30–40% confluence 1 day prior. Every 2 days, the culture medium was refreshed for 12 days.

To produce rCHO cells expressing CM21D and CM21SD, using the RMCE/Bxb1 system, a co-transfection procedure was executed on the CHO-K1 cell platform. This process utilized the Bxb1 recombinase vector (Addgene plasmid #51552) (Zhu et al. 2014) and the RMCE donor plasmid, which encodes CM21D and CM21SD in a 1:3 ratio. Furthermore, by utilizing the RMCE/Bxb1 system, the rCHO cells that co-express CM21D and hrsACE2 as secretory recombinant proteins were generated. To achieve this, a co-transfection procedure was performed using a Bxb1 recombinase vector and RMCE donor plasmid, which encodes CM21D (at a 1:3 ratio) on clonal cells expressing hrsACE2 within the upstream region of the *S100A* gene cluster (Ghanbari et al. 2023). The co-transfection specifically retargeted a pre-existing Bxb1 landing pad integrated into the genome, which had been developed in our ongoing study. A day prior to transfection, CHO-K1 cell platforms were seeded at a density of 6 × 10^4^ cells/well in 24-well plates, and the Lipofectamine 3000 transfection reagent (Thermo Fisher, Invitrogen™, Waltham, MA, USA) was used according to the manufacturer’s protocol. Post-transfection, the cells were relocated into 6-well tissue culture plates after 72 h. On the 7th day following transfection, the cells underwent selection with 5 μM ganciclovir (GCV).

### 5′/3′ junctions and out-out PCR

Following the expansion of stable colonies, genomic DNA was isolated using a genomic DNA extraction kit (Qiagen, Hilden, Germany) according to the manufacturer’s protocol and PCR targeting the 5′/3′ junctions was conducted on the pool of stable cells. Single-cell cloning was carried out via limiting dilution in the 96-well plate, maintaining a density of one cell per well. After 6 days, single colonies were transferred to 24-well plates. In the case of rCHO cells, EGFP-expressing single clones were transitioned to 24-well plates, and expression of EGFP was subsequently monitored utilizing a fluorescence microscope. The genomic DNA extraction is performed using the cell lysates procedure (Pourtabatabaei et al. 2021). Single-cell pellets were treated with 20 μL of 0.2 M NaOH and incubated at 75 °C for 10 min. The reaction was then neutralized by adding 180 μL of 0.04 M TRIS–HCl (pH = 7.8). For each corresponding 5′/3′ junction PCR reaction, 2.5 μL of each lysate was used as a template. The PCR conditions were set as follows: 95 °C for 5 min; 30 × : 95 °C for 30 s, 58 °C for 30 s, 72 °C for 50 s, and 72 °C for 10 min. The PCR was facilitated using the 2X PCR Master Mix Red (Ampliqon, Odense M, Denmark) with specific primers (Supplemental Table S1). The DNA was extracted from 5′/3′ junction PCR-positive clones using a genomic DNA extraction kit, following the manufacturer’s protocol (Qiagen, Hilden, Germany); then, out-out PCR was carried out using (2X SuperAdd Taq Master, Addbio Inc., Daejeon, South Korea) according to the following PCR program: 95 °C for 5 min; 32 × : 95 °C for 30 s, 60 °C for 30 s, 72 °C for 3 min and 72 °C for 10 min using specific primer (Supplemental Table S1). PCR products were visualized on a 1% agarose gel.

### Flow cytometry

1 × 10^5^ cells from each EGFP-expressing clone were collected and resuspended in phosphate-buffered saline (PBS). These cells were then analyzed on a flow cytometer (IndiaMART, Sysmex CyFlow Counter, Jammu Kashmir, India) to quantify the EGFP + cells. Non-transfected CHO-K1 cells were used as the negative control for setting the gating.

### Growth profile and viability curve determination

For the analysis of growth profiles and cell viability, the clonal cells and pool cells, along with control groups, were inoculated at a density of 2.5 × 10^4^ cells per well in 12-well plates. The density of viable cells and viability were then quantified daily over a period of 10 days. Each day, assessments were conducted on two wells.

### Copy number analysis using quantitative real-time PCR (qRT-PCR)

The copy numbers of the *TK* gene (for thymidine kinase) and *EGFP* gene were determined via relative qRT-PCR, performed on selected genomic DNA samples of CHO-K1 cell platforms and rCHO-K1 cells using *TK* and *GFP* genes specific primers, respectively, and *ACTB* (for β-actin) as the reference gene (Supplemental Table S1). The calibration was conducted in relation to cells known to harbor a single copy of the *TK* and *GFP* genes. Reaction mixtures, prepared in duplicate, included 2 × Real-Time PCR Master Green (Ampliqon, Odense M, Denmark), and the PCR was performed with the ABI 7500 Real-time PCR System (Thermo Fisher, Applied Biosystems™, Waltham, MA, USA). All primers were empirically validated through melting curve analysis and agarose gel electrophoresis. Amplification was carried out under the following conditions: 95 °C for 15 min, 40 × :95 °C for 20 s, 60 °C for 35 s. The evaluation of the copy number was based on the threshold cycle, Ct, and was quantified using the 2^−ΔΔct^.

### RNA extraction, cDNA synthesis, and qRT-PCR

Total RNA was extracted from rCHO-K1 clones expressing CM21D, CM21SD, and CHO-K1 cell platform using a Total RNA Extraction kit (Parstous, Mashhad, Iran), followed by DNase treatment according to the manufacturer’s protocol. RNA quality was evaluated using a NanoDrop spectrophotometer at an absorbance ratio of 260/280 nm. Subsequently, 500 ng of total RNA underwent reverse transcription using the cDNA Synthesis kit (Parstous, Mashhad, Iran), following the manufacturer’s recommended protocol. The quantification of miR-21 expression was carried out using the stem-loop method. Reaction mixtures, prepared in duplicate, included 2 × Real-time PCR Master Green (Ampliqon, Odense M, Denmark), and the PCR was performed with the ABI 7500 Real-time PCR System (Thermo Fisher, Applied Biosystems™, Waltham, MA, USA). qRT-PCR performed under the following conditions: 95 °C for 10 min, 40 × :95 °C for 15 s, 60 °C for 1 min. The U6 snoRNA gene was employed as the reference gene for the normalization of miR-21, and the CHO-K1 cell platform was used as calibrator cells. The expression levels of *Pdcd4*, *Trim33*, and *Atp11b* (identified as target genes of miR-21) were quantified under the following conditions: 95 °C for 10 min, 40 × :95 °C for 15 s, 60 °C for 1 min. *ACTB* served as the reference gene for the normalization of mRNA expression levels. CM21SD Expression rCHO-K1 cells were used as calibrator cells to analyze miR-21 target genes. The expression level of the CM21D and CM21SD was calculated under the following conditions: 95 °C for 15 min, 40 × :95 °C for 15 s, and 62 °C for 1 min. The U6 snoRNA gene was employed as the reference gene for the normalization. The expression of miR-21, CM21D, CM21SD, and all target genes were evaluated using specific primers (Supplemental Table S1). All primers were empirically validated through melting curve analysis and agarose gel electrophoresis. Quantification was performed based on the threshold cycle (Ct) and calculated using the 2^−ΔCt^ method to evaluate CM21D and CM21SD expression levels and the 2^−ΔΔCt^ method for the analysis of miR-21 and miR-21 target genes.

### Enzyme-linked immunosorbent assay

The impact of CM21D expression on productivity was assessed by utilizing a pool of cells co-expressing hrsACE2 and CM21D. Non-retargeted hrsACE2-expressing CHO-K1 clonal cells were employed as a control group, while non-transfected CHO-K1 cells served as the negative control. Each group of cells was seeded at 30% confluence in T-25 cell culture flasks for 6 days. The resulting cell culture supernatant was collected, and the concentration of secreted hrsACE2 was quantified using the Human ACE-2 DuoSet ELISA kit (Bio-Techne, Minneapolis, MN, USA) following the manufacturer’s protocol.

### Statistical analysis

All statistical analyses were performed using GraphPad PRISM version 8.0.2 (GraphPad, Boston, MA, USA). To compare differences between the control and test groups and assess the statistical significance of these variations, *t*-tests were employed with a predetermined significance level of 0.05 (*p*-value < 0.05). The graphical representations of the data depict the mean ± standard deviation (SD).

## Results

### Comparison of knock-in efficiency of RNP and plasmid delivery systems

**Fig. 1:**
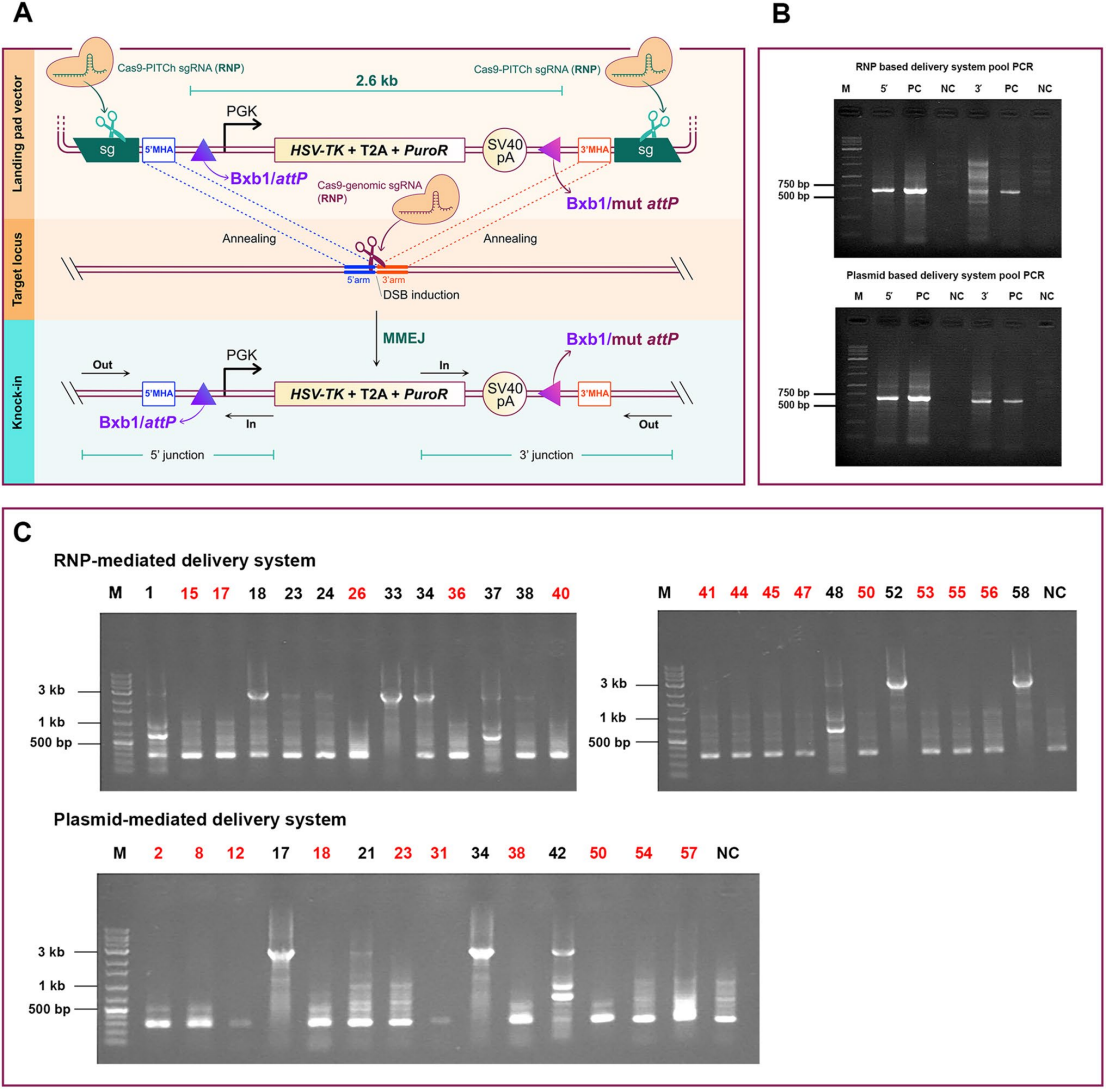
Knock-in process and agarose gel electrophoresis of 5′ and 3′ junction PCR products in stable cell pools and out-out PCR. **a** Schematic illustration of PITCh sgRNA, genomic DNA cut sites, the landing pad donor vector, and the knock-in process mediated by the microhomology arms. **b** The agarose gel electrophoresis results for 5′ and 3′ junction PCR products in stable cell pools transfected using RNP and plasmid-based delivery systems. The anticipated PCR product for the 5′ junction PCR and 3′ junction PCR are 666 bp and 622 bp, respectively. **c** The agarose gel electrophoresis results for the out-out PCR on 3′/5′ positive junction clones of RNP and the plasmid-mediated delivery system. The anticipated sizes of the PCR products for the out-out PCRs were 246 bp and 2980 bp. M corresponds to the 1 kb DNA marker, PC stands for the positive control, and NC represents the negative control

To compare the CRIS-PITCh mediated knock-in efficiency of RNP and plasmid-based delivery systems, the upstream site of the *S100A* gene cluster—which has been identified as an active transcriptional locus—was addressed, with the aim to create the CHO-K1 platform cell for maintained protein expression (Ghanbari et al. 2023; Rahmani et al. 2023). CHO-K1 cell platforms were generated using CRIS-PITCh-mediated targeting with both plasmid and RNP-based delivery systems. A PITCh donor plasmid was utilized, with 30 bp 5′ and 3′ microhomology arms flanking the cleavage site of the sgRNA, which was designed in our previous study (Ghanbari et al. 2023). In the context of the plasmid-based delivery system of CRIS-PITCh, an all-in-one vector and the PITCh donor plasmid were co-transfected into the CHO-K1 cells. In the RNP-based delivery system, PITCh and genome-targeting sgRNAs were first in vitro-synthesized and visualized on a 2% agarose gel (Supplemental Fig. S1). Two RNP complexes were then utilized. The first complex, containing PITCh sgRNA, induced a double-strand break in the PITCh donor plasmid, facilitating in vivo plasmid linearization. The second one, containing genome-targeting sgRNA, was responsible for creating a double-strand break in the genomic DNA (Fig. 1a). The PITCh donor plasmid was transfected into the cells using Lipofectamine 3000, which took place 4 h prior to the CRISPRMAX™ transfection. Stable cell pools were generated following puromycin selection. After cell pool expansion and genomic DNA extraction, a 5′/3′ junction PCR was performed to verify the knock-in process. According to the gel electrophoresis data, the 5' junction band size is 666 bp, and the 3′ band size is 622 bp (Fig. 1b). After confirming stable cell pools, single-cell cloning of the stable cell pools was performed using limiting dilution. The efficiency was measured by 5′/3′ junction and out-out PCRs. Utilizing both RNP and plasmid-based delivery systems, we recovered and analyzed 58 and 60 single clones, respectively, using 5′/3′ junction PCR. From these, 23 clones (39.65% of the total) from the RNP system and 14 clones (23.33% of the total) from the plasmid-based system tested positive for the 5′/3′ junction (Supplemental Fig. S2 and S3). The out-out PCR process yielded a product indicating 2980 bp. A band indicating 246 bp in the out-out PCR indicated either mono-allelic integration or non-integrants. Upon further analysis, it was found that 11 of the junction PCR-positive clones in the RNP-based delivery system (44% of the clones) and 4 of the junction PCR-positive clones in the plasmid-based delivery system (28.5% of the clones) were successfully targeted with the entire PITCh donor plasmid. Notably, five clones (numbered 33, 37, 48, 52, and 58) in the RNP delivery system and two (numbered 17 and 34) in the plasmid-based delivery system exhibited only a 2980 bp band, suggesting biallelic integration. In addition to the 2980 bp band, six clones (numbered 1, 18, 23, 24, 34, and 38) in the RNP delivery system and two clones (numbered 21 and 42) in the plasmid-based delivery system exhibited the 246 bp band, suggesting mono-allelic integration (Fig. 1c).

### Copy number analysis and random integration effect

**Fig. 2:**
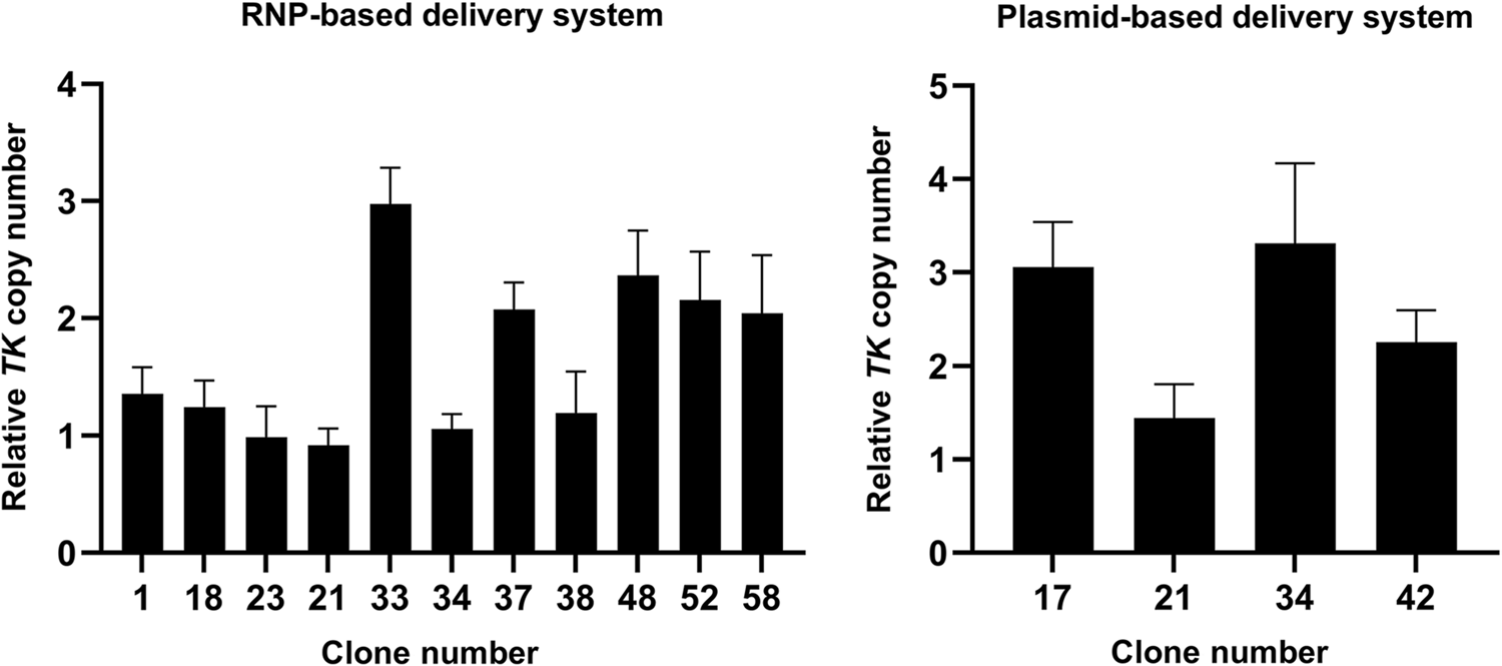
The relative copy number of *TK* in positive out-out PCR clones. The error bars illustrate the standard deviations based on technical replicates (*n* = 3)

To discern the implications of the RNP delivery system on random integration and off-target effects, we estimated the *TK* transgene copy number in clonal cells. Under the RNP-based delivery system, a set of 11 single-cell clones that tested positive in the out-out PCR were evaluated via qRT-PCR. Six of these clones, which indicated mono-allelic integration of the landing pad cassette in the out-out PCR results, represented a single copy in the quantitative PCR analysis (numbered 1, 18, 23, 24, 34, and 38). Concurrently, bi-allelic integration was observed in five clones in the out-out PCR but aligned with a two-copy outcome in the relative qRT-PCR in four clones (37, 48, 52, and 58). Only in clone 33, we observed a copy number of 3 for the landing pad. Four single-cell clones that tested positive in the out-out PCR were subjected to qRT-PCR analysis in the study involving a plasmid-based delivery system. Our findings revealed that one of the two clones that indicated mono-allelic integration of the landing pad cassette in the out-out PCR results exhibited mono-allelic integration (numbered 21). Furthermore, two clones showed bi-allelic integration during the out-out PCR analysis, but one matched a two-copy outcome in the subsequent qRT-PCR (numbered 42). In accordance with our observations, a substantial number of clones (numbered 17 and 34) within the plasmid-based delivery system manifested random integration, a phenomenon we attributed to off-target effects (Fig. 2). This random integration frequency was one clone among the 11 tested clones in the RNP-based delivery system, thereby indicating a superior degree of specificity associated with this method. A mono-allelic clonal cell, harboring one copy of the PITCh donor plasmid in the RNP-based delivery system, was selected for subsequent steps (numbered 34).

### Designing miR-21 sponge

**Fig. 3:**
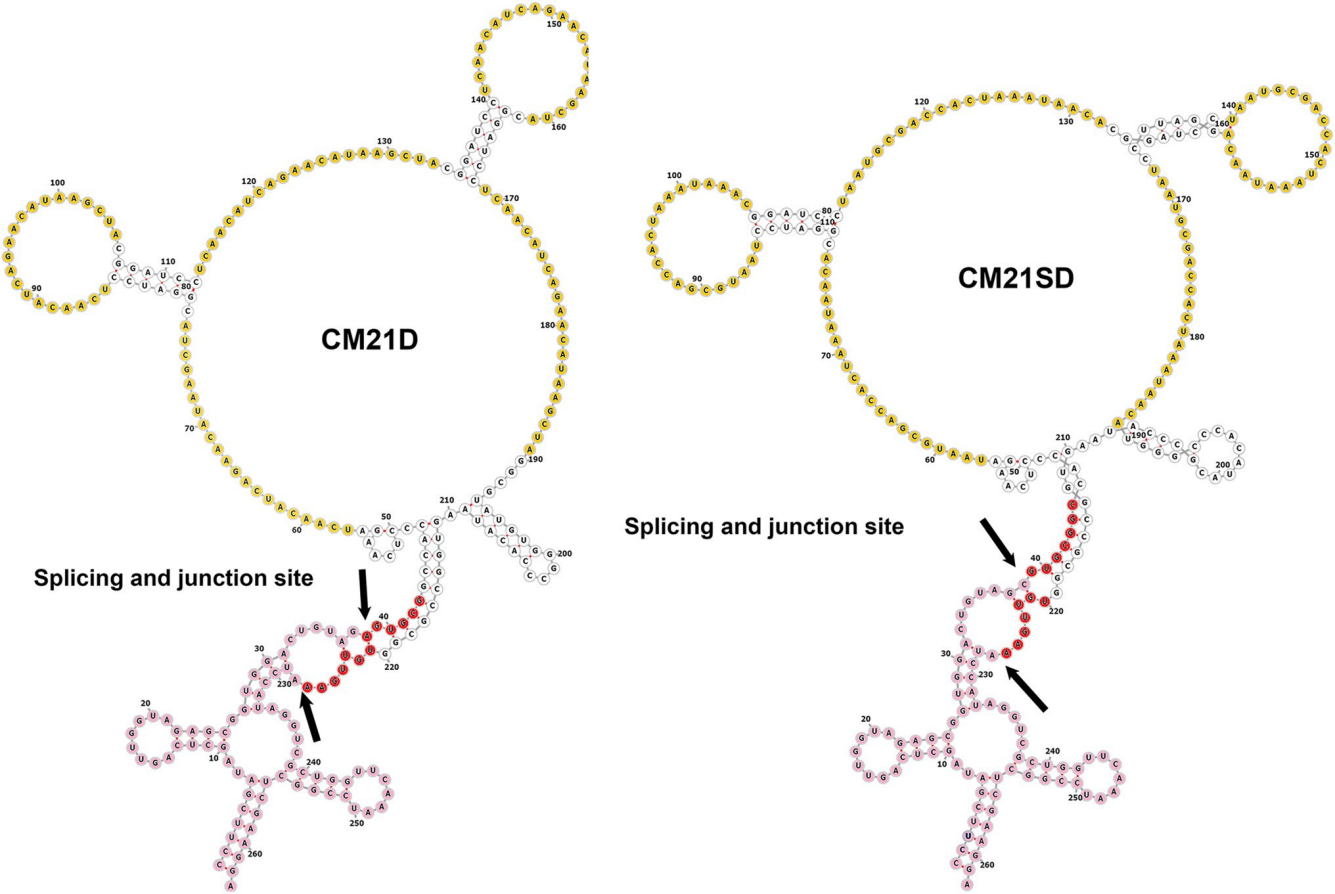
Schematic overview of circular miR-21 decoy and circular miR-21 scramble decoy generation via a tRNA-splicing mechanism. Using MXfold2, the secondary structure of *Drosophila* Tyr-tRNA-CM21D and CM21SD was predicted. In the schematic overview, tRNA is represented in purple, miR-21 bulged binding sites, and miR-21 scrambled binding sites in yellow, and black arrows denote splice sites

Bayat et al. (2023) designed a CM21D with three bulged binding sites; they found that this structure could reduce tumor volume in the GBM rat model. They used random integration to express RNA decoys in U87 and C6 cells (Bayat et al. 2023). Therefore, to create rCHO cells that express CM21D, which is a safe and high-producer cell (Sanny et al. 2006), we designed a CM21D with five bulged binding sites. We used the *Drosophila* Tyr-tRNA gene, CR31905, to generate a circular sponge structure, which splices its intron into a circular shape in mammals (Bayat et al. 2023). We replaced the intron of the gene with CM21D and CM21SD. The secondary structure of the recombinant tRNA gene, CM21D and CM21SD, was designed and predicted by the MXfold2 online tool (Fig. 3).

### Creating RNA decoys expressing clonal cells using the RMCE system

**Fig. 4:**
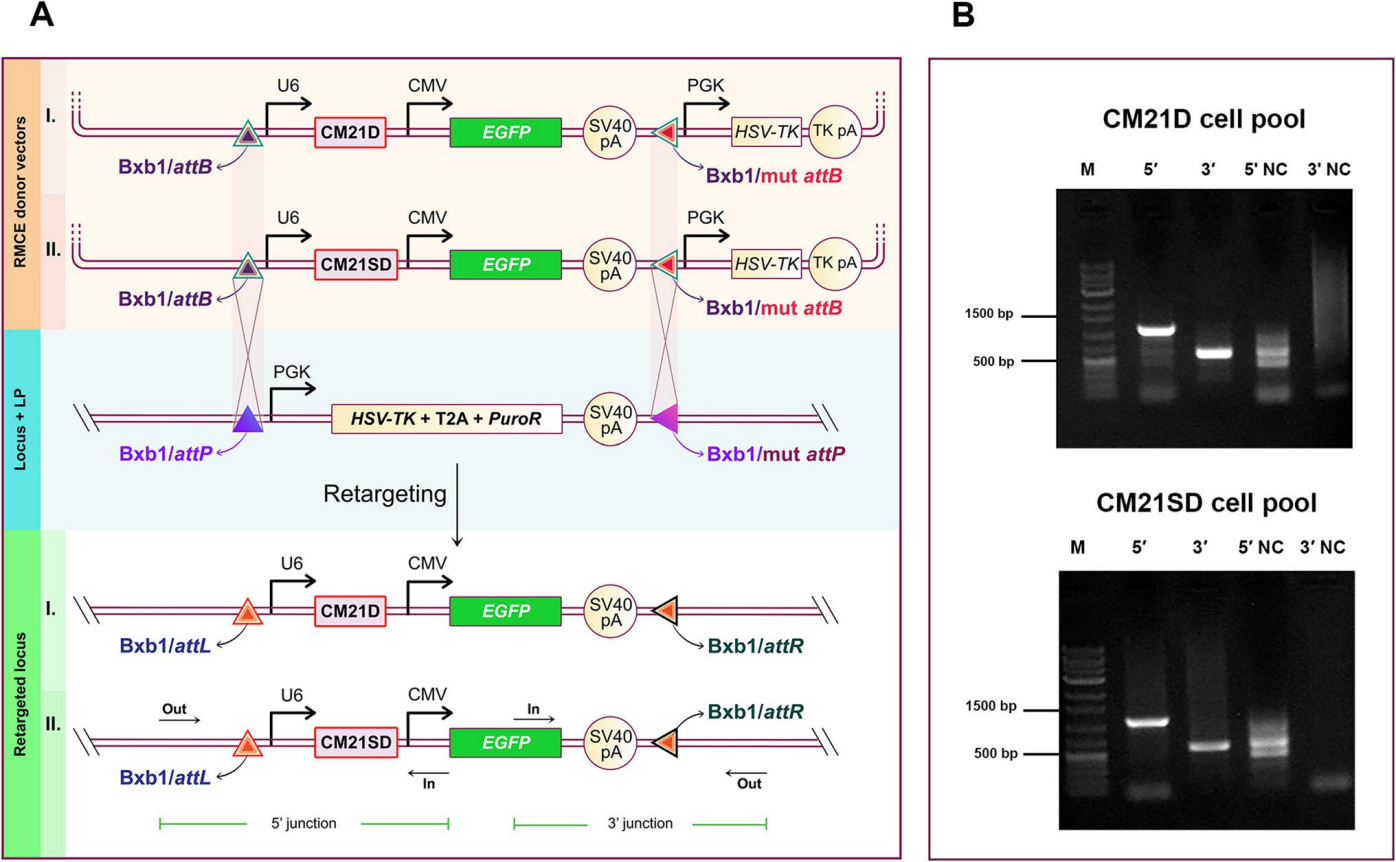
RMCE/Bxb1 integration process and agarose gel electrophoresis for 5′/3′ junction PCR on the cell pool co-transfected with CM21D and CM21SD donor vectors, along with Bxb1 integrase after GCV selection. **a** Schematic illustration of CM21D and CM21SD RMCE donor vectors and RMCE/Bxb1 targeted integration process. **b** The anticipated PCR fragment sizes were approximately 1360 bp for the 5′ junction and 646 bp for the 3′ junction PCRs. M corresponds to the 1 kb DNA marker, and NC represents the negative control

One of the mono-allelic integrants (*TK* single copy clonal cell) created using the RNP-based delivery system was chosen (clone 34). The CM21D and CM21SD encoding vectors, flanked by the *attB* recognition sequence, were used as RMCE donor plasmids. The RMCE donor plasmids and the Bxb1 recombinase vector were used to co-transfect the selected clone. Following transfection, cells were subjected to GCV negative selection (Fig. 4a). 5′/3′junction PCR was performed on the emerging cell pool (Fig. 4b). After the limiting dilution process, the emerging colonies were inspected under a fluorescence microscope. Genomic DNA was then extracted from the cells expressing EGFP. Out of the 10 tested clones, 8 were positive for the 5′/3′ junction in CM21D, and 6 were positive in CM21SD-retargeted clones. The PCR fragment sizes were approximately 1360 bp for the 5′ junction and 646 bp for the 3′ junction PCRs (Supplemental Fig. S4).

### Validation of clonal cells by flow cytometry

The 5′/3′ junction positive clones (8 clones related to CM21D and 6 clones related to CM21SD) underwent flow cytometry analysis, with non-transfected CHO-K1 cells as the negative control. The data from the flow cytometry revealed that 7 clones of CM21D (numbered 1, 3, 5, 6, 7, 9, and 10) and 1 clone of CM21SD (numbered 4) expressed EGFP in ≥ 99% of cells. Clones 3, 6, and 9 were chosen for growth profile and viability analysis (Supplemental Fig. S5).

### Growth profile and viability of the CM21D and CM21SD expression clonal cells

**Fig. 5:**
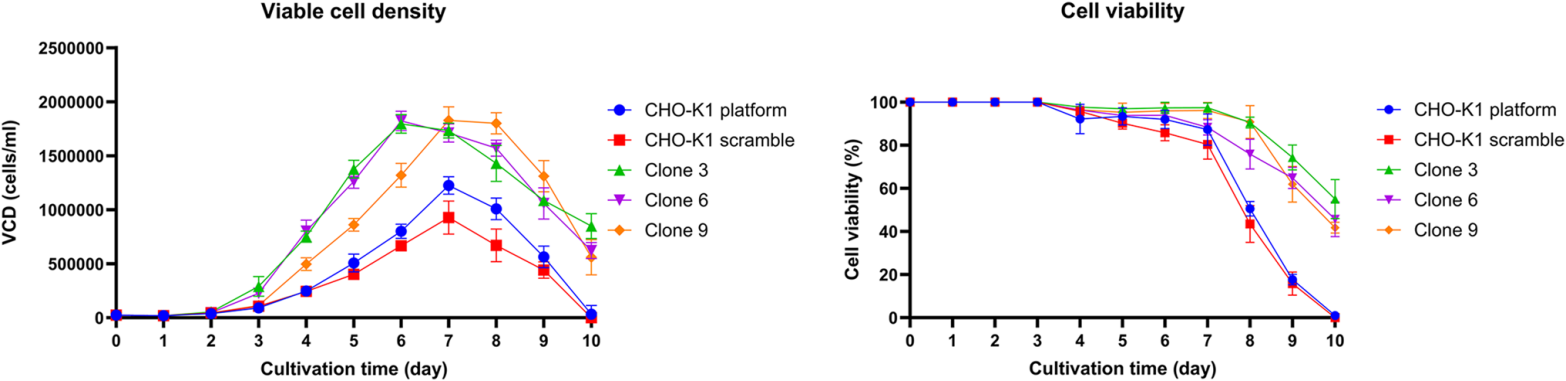
Viable cell density and cell viability curves indicated the impact of CM21D expression on CHO cells during a 10-day batch culture. Each data point on the growth and viability curves corresponds to a specific day. Notably, the error bars represent the standard deviation based on technical replicates (*n* = 3)

After creating rCHO-K1 cells expressing CM21D, we observed a significant increase in the growth rate of CHO cells. This surprising phenomenon occurred during the cell culture process, which is beneficial for industrial production. To further investigate, we compared the viable cell density (VCD) and viability of CM21D-expressing clonal cells (clones 3, 6, and 9) with that of CM21SD-expressing and CHO-K1 platform clonal cells over 10 days. The growth profile curve indicated that the growth rate in CM21D-expressing clonal cells increased approximately 1.3-fold in batch culture, changing to 0.90 1/d, rather than 0.70 1/d for CM21SD-expressing clonal cells, and cell viability remained above 40% on day 10, unlike the control groups, where the viability dropped to zero (Fig. 5). The growth rate was determined from the following formula: $\mu =\frac{{\varvec{ln}}\left(\frac{{{\varvec{N}}}_{{\varvec{t}}}}{{{\varvec{N}}}_{0}}\right)}{\Delta {\varvec{t}}}\bullet 24\text{ h}$.*µ* = growth rate [1/d]Δt = hours of growth [h]*N*_0_ = number of cells seeded*N*_1_ = number of cells harvested

### Relative qRT-PCR *to mono*-allelic clonal cell selection

**Fig. 6:**
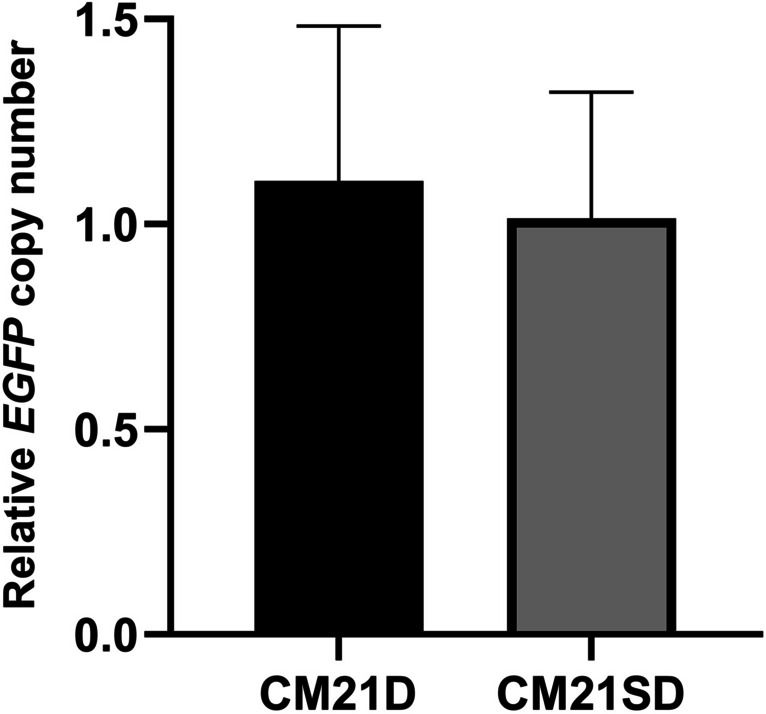
Relative qRT-PCR is used to determine the *EGFP* copy number in retargeted clonal cells. The error bars correspond to the standard deviations observed in technical replicates (*n* = 3)

The copy numbers of the CM21D and CM21SD cassettes were determined using relative qRT-PCR, which amplified the *EGFP* gene. The clonal cell carrying one copy of *EGFP* served as the calibrator cell. It was found that both clones contained one copy of *EGFP* (Fig. 6).

### Determination of the expression level of miR-21 in the clonal cells expressing CM21D and CM21SD

**Fig. 7:**
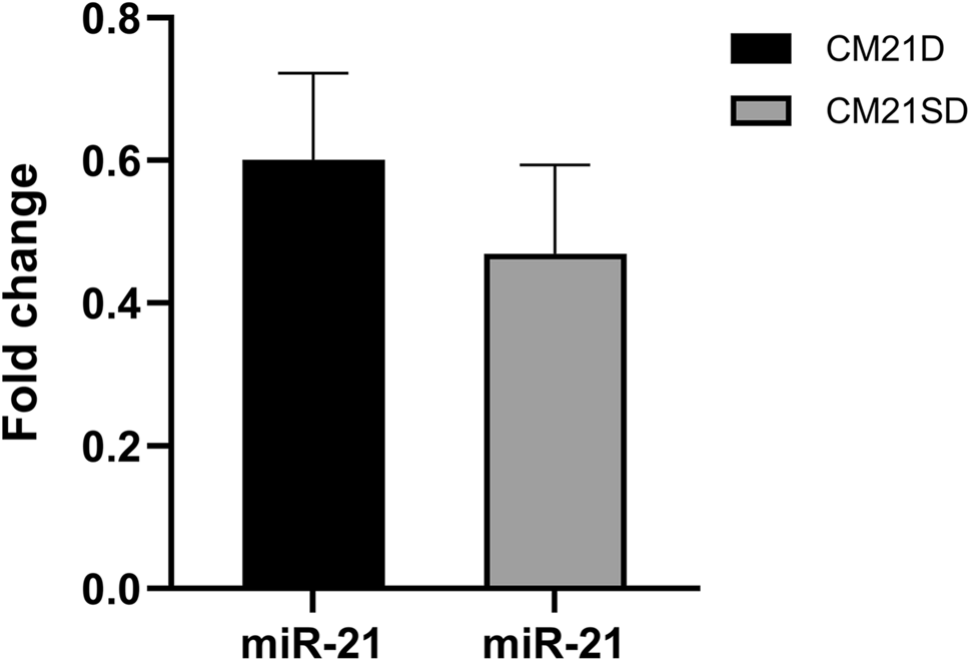
The miR-21 expression equivalence verification. qRT-PCR was performed to compare miR-21 expression levels between CM21D and CM21SD expressing clonal cells. MiR-21 expression levels were equivalent in these clonal cells (*p* < 0.05). The error bars correspond to the standard deviations observed in technical replicates (*n* = 3)

Relative qRT-PCR was utilized to determine the expression level of miR-21 in CM21D and CM21SD expressing cells to prove that the expression level of miR-21 is not significantly different in expressing CM21D and CM21SD clonal cells. The CHO-K1 cell platform served as the calibrator cell. Results showed that the level of expression in both clonal cells is approximately equal (Fig. 7).

### Confirmation of the structure and expression of RNA decoys in rCHO cells

**Fig. 8:**
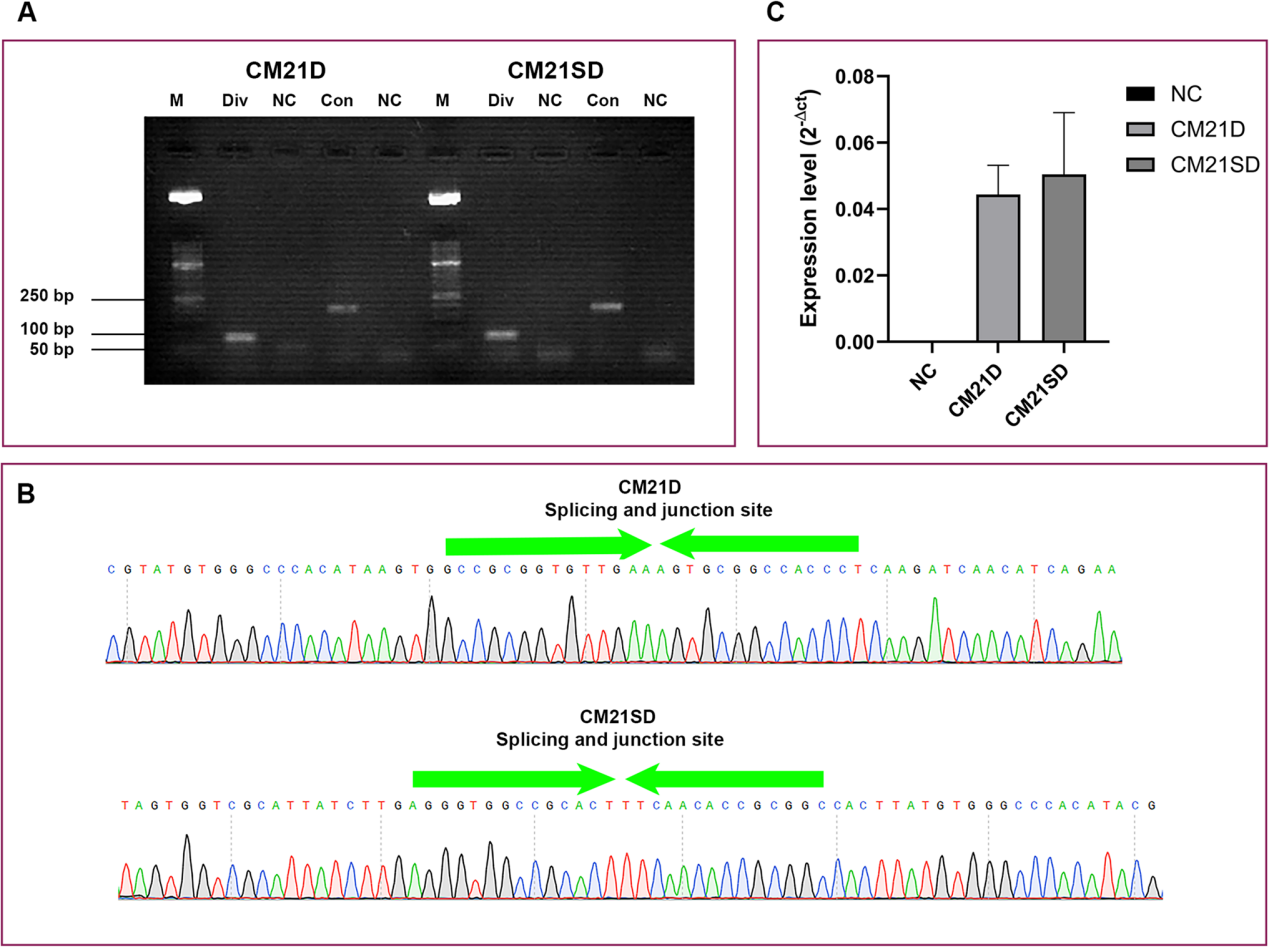
Validating the expression and circularization of CM21D and CM21SD. **a** Expression and circularization of CM21D and CM21SD were first confirmed by RT-PCR using divergent and convergent primer sets in CM21D and CM21SD expressing clonal cells. The agarose gel electrophoresis of expected fragment size for divergent and convergent PCR products were 84 and 194 bp, respectively. Div represents the divergent. Con, Convergent. NC, negative control, and M, 50 bp DNA ladder. **b** Sanger sequencing results related to divergent primer set PCR products. **c** Expression level of CM21D and CM21SD was measured using qRT-PCR (based on the formula 2 ^(−ΔCt^.^)^). The error bars correspond to the standard deviations observed in technical replicates (*n* = 3)

The formation of circular structures in CM21D and CM21SD was examined using reverse transcription PCR. This process utilized a set of convergent and divergent primers (Supplemental Table S1). The convergent primer confirmed the linear structure (192 bp), while the divergent primer confirmed the circular structure (84 bp) (Fig. 8a). To further validate these findings, the divergent PCR products were sequenced using Sanger sequencing (Fig. 8b). The observation of GFP expression in these two clonal cell lines serves as empirical evidence for the in vivo expression of CM21D and CM21SD. To confirm the generation of CM21D and CM21SD in the expressing clonal cells, qRT-PCR was performed to assess the expression level of the target gene. The calculation was based on the formula 2 ^(−ΔCt)^, where ΔCt represents the difference between the Ct value of any CM21D or CM21SD and the Ct value of the reference gene (Fig. 8c).

### Analysis of RNA decoys functionality with qRT-PCR on target genes

**Fig. 9:**
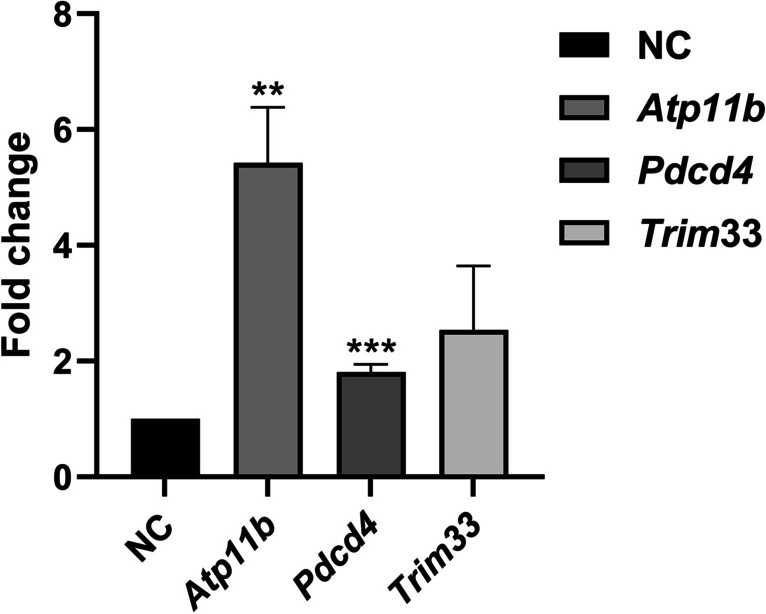
Expression level of miR-21 target genes. The expression of three target genes of miR-21 was measured using qRT-PCR. The *Atp11b* and *Pdcd4* genes were significantly expressed in the presence of CM21D (*p* < 0.05). The error bars correspond to the standard deviations observed in technical replicates (*n* = 3)

MicroRNA-21 (miR-21) is known to target and downregulate specific genes. We assessed the expression levels of *Pdcd4*, *Trim33*, and *Atp11b*, which were identified as potential miR-21 target genes (Maccani et al. 2014) in clonal cells expressing CM21D using qRT-PCR with gene-specific primers (Supplemental Table. S1). The gene *ACTB* was utilized as a reference gene. Clonal cells that express CM21SD acted as the calibrator cells. The data we gathered indicates that the introduction of CM21D reinstates the expression of these genes, but in the *Atp11b* and *Pdcd4* genes, the reinstatements were significant statistically (Fig. 9).

### Analysis of the productivity, viable cell density, and viability of recombinant protein in rCHO cells expressing CM21D

**Fig. 10:**
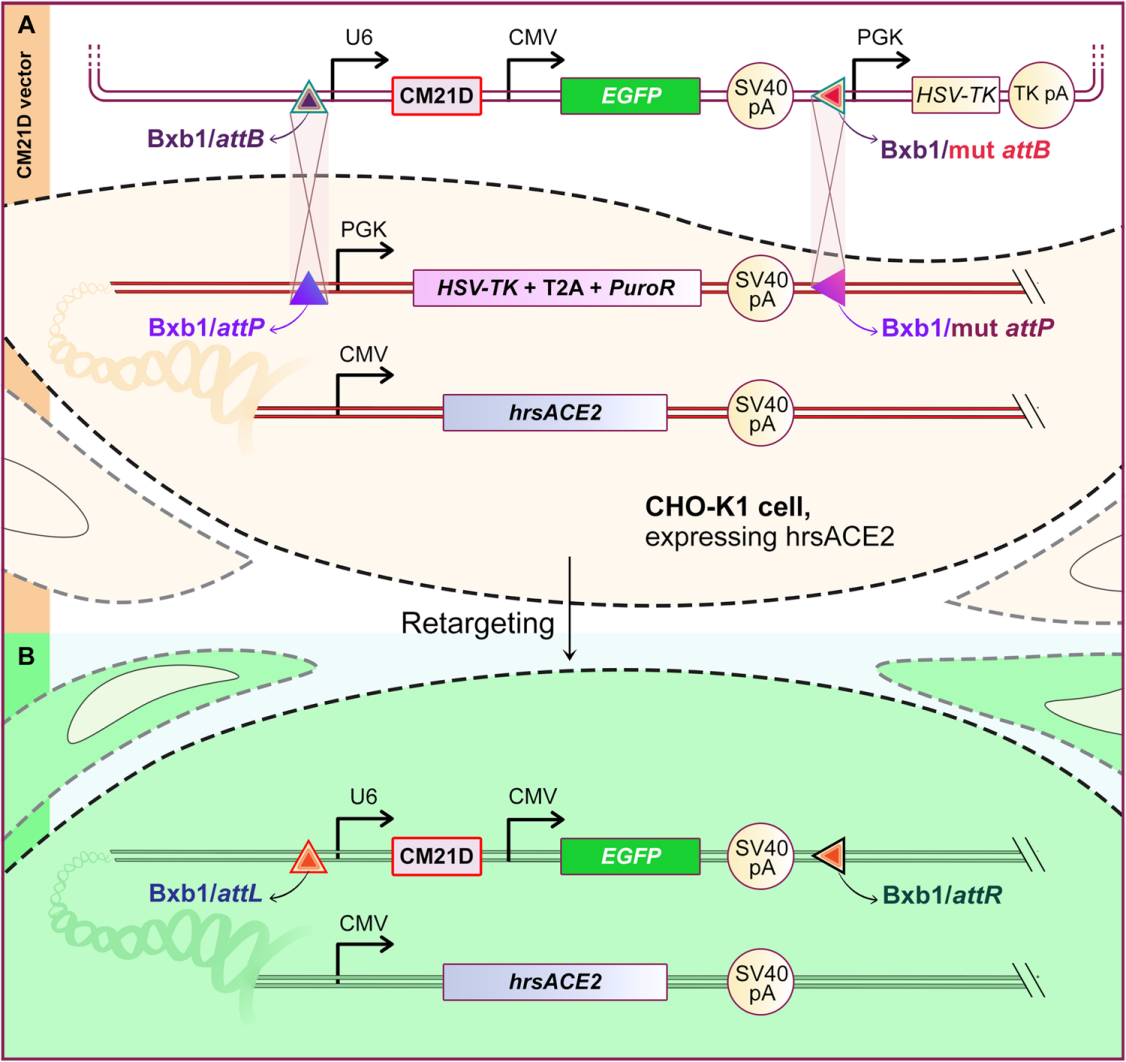
Bxb1/RMCE retargeting process. Schematic illustration of RMCE/Bxb1 retargeting in CHO-K1 clonal cells that express hrsACE2 harboring pre-existing landing pad

**Fig. 11:**
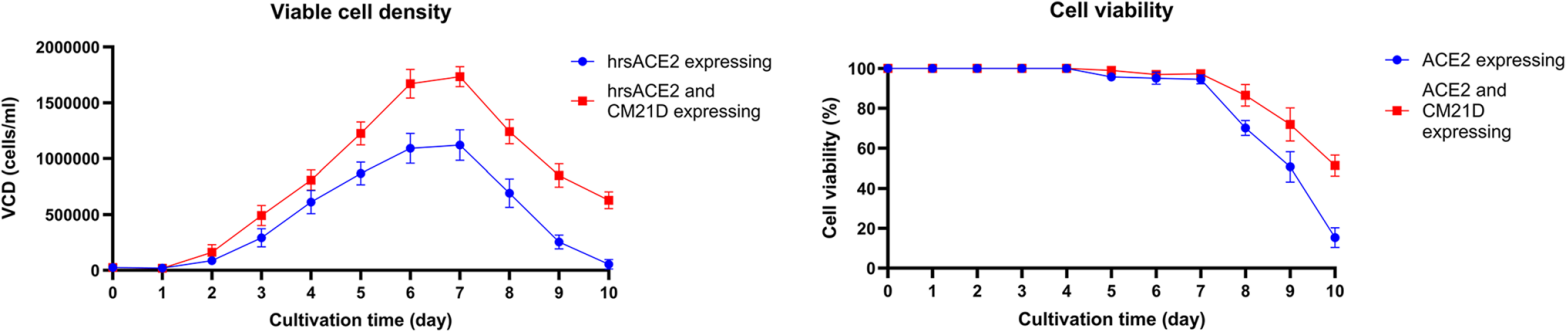
The viable cell density and cell viability curves reveal the influence of CM21D expression on CHO cells expressing hrsACE2 over a 10-day batch culture. Each data point on these curves corresponds to a specific day. Notably, the error bars represent the standard deviation based on technical replicates (*n* = 3)

To investigate the impact of CM21D expression on recombinant protein productivity, we retargeted the CM21D cassette in CHO-K1 clonal cells that express hrsACE2 as secretory recombinant proteins. This retargeting was performed within an integrated pre-existing landing pad, which is essential for the RMCE/Bxb1 system (Fig. 10). After GCV selection, the cell pool underwent flow cytometry analysis, with non-transfected CHO-K1 cells serving as the negative control. The data from the flow cytometry revealed that the cell pool expressed 84% EGFP. To validate the production of CM21D in the cell pool, qRT-PCR was performed to assess the expression level of the target gene. The quantification was determined using the formula 2 ^(−ΔCt)^ (Supplemental Fig. S6). The productivity of hrsACE2 using the Human ACE-2 DuoSet ELISA kit was measured (Supplemental Fig. S7). Cell pool co-expressing hrsACE2 and CM21D, as well as the clonal cells expressing hrsACE2, were utilized. Additionally, the non-transfected CHO-K1 cells served as the negative control group. These cells were seeded in a T-25 cell culture flask. After a period of 6 days, the cell culture supernatant was collected from each group. In the CM21D-expressing pool cells, the concentration of hrsACE2 was 7.56 µg/ml, whereas in the control group, it measured 3.67 µg/ml. Notably, the CM21D-expressing pool cells exhibited a significant 2.06-fold increase in hrsACE2 titer (Supplemental Fig. S7). Through the utilization of the growth profile curve, the growth rate of the cell pool co-expressing hrsACE2 and CM21D, as well as that of the clonal cells expressing hrsACE2, was calculated over 10 days. The growth rate of the cell pool co-expressing hrsACE2 and CM21D was increased to 0.90 1/d, compared to 0.82 1/d for the clonal cells expressing hrsACE2. On day 10, cell viability remained above 50%, in contrast to the control group, where viability decreased to 15% (Fig. 11).

## Discussion

The therapeutic promise of the miR-21 sponge, particularly in the treatment of glioblastoma, has been significantly highlighted in research findings (Monfared et al. 2019; Bayat et al. 2023). Considered safe and highly productive, CHO cells have been examined in relation to miR-21, which has been shown to have an anti-apoptotic role in other mammalian cell lines (Meleady et al. 2011; Moshfeghnia 2023). The overexpression of miR-21 had a slight detrimental impact on CHO cell growth and viability during late exponential-phase culture (Belliveau et al. 2024). Given the conserved sequence of miR-21 between *Homo sapiens* and the hamster *Cricetulus griseus*, we designed a miR-21 sponge with 5 bulge sequences to inhibit miR-21. We employed the CRIS-PITCh combined with the RMCE approach as a targeted integration method. Such techniques make it easier to insert the transgene in a predetermined “hot spot” with a defined copy number and reproducible transgene targeting, resulting in more consistent and stable gene expression (Rehberger et al. 2013; Zhou et al. 2016; Hamaker and Lee 2018). As part of our study, we proceeded by creating a CHO-K1 cell platform, which is characterized by the presence of a landing pad incorporating the *attP* recognition sequence utilizing CRIS-PITCh technology. Despite the fact that the CRIS-PITCh method used for creating the CHO-K1 cell platform is associated with relatively low knock-in efficiency and high off-target effects (Guo et al. 2023; Rahmani et al. 2023), according to Kim et al. (2014) and Naeem et al. (2023), the RNP-based delivery system can improve knock-in efficiency along with the reduction of off-target effects in the CRISPR/Cas9 system (Kim et al. 2014; Naeem and Alkhnbashi 2023). We adopted both the RNP-based approach and the plasmid-based system for the delivery of the CRIS-PITCh method in CHO-K1 cells and then compared the knock-in efficiency as well as off-target effects across these two systems. The 5′/3′ junction PCR findings showed that the knock-in efficiency had increased by 1.7-fold. Furthermore, real-time PCR indicated that an ignorable level of random integration (off-target effect) exists in the RNP-based delivery system as opposed to the plasmid-based delivery method. In the second step, we selected a CHO-K1 clonal cell platform with a single copy landing pad and no random integration for the generation of recombinant CHO-K1 expressing CM21D and CM21SD with 5 bulge sequences. As suggested by Inniss et al (2017), the RMCE/Bxb1 system offers greater precision in the creation of CHO cell clones with high-fidelity cassette exchange events (Inniss et al. 2017). It was hypothesized that the insertion of the *HSV-TK* cassette into the RMCE vector’s backbone inhibits any undesired recombination events. Following Bateman et al.’s (2006) recommendations, we arranged the *att* sites in a reverse orientation to prevent the landing pad from exchanging with the plasmid backbone instead of the gene of interest (GOI) cassette. Our real-time PCR results showed a single copy number of CM21D and CM21SD in the chosen transfected clonal cells, which is consistent with the findings of the previously cited research (Ghanbari et al. 2023). We conducted qRT-PCR to assess the expression level of CM21D and CM21SD. Remarkably, we observed statistically equal expression of these decoys. This finding dispels any ambiguity regarding whether subsequent changes in the cell are attributable to expression differences between these two structures. To confirm the formation of circular structures in CM21D and CM21SD, the PCR products resulting from reverse transcription PCR were sequenced using Sanger sequencing. The results revealed that circular structures were indeed formed due to the existing *Drosophila* tRNA splicing mechanism (Bayat et al. 2023). Subsequently, we assessed the functionality of CM21D following its expression in the CHO-K1 cell platform. An analysis was conducted on the expression of miR-21 target genes in comparison with CM21SD-expressing clonal cells.

The data indicated that there was an increase in the gene expression of *Pdcd4*, *Trim33*, and *Atp11b* compared to the cells expressing CM21SD. This increase was statistically significant for *Atp11b* and *Pdcd4* genes, according to the data analysis. The findings of our investigation on the upregulation of miR-21 target genes in the presence of CM21D are in agreement with those of Bayat et al. (2023). Therefore, it can be concluded that CHO-K1 cells are proficient in the production of this RNA decoy.

With the aim of CHO engineering by CM21D, during the process, we noticed a 1.3-fold increase in growth rate compared to CM21SD-expressing clonal cells. In order to shed light on this phenomenon, we comprehensively reviewed the existing literature on the role of miRNAs in CHO cell behavior. Using a recombinant, Epo-Fc generating CHO cell line, the researchers in Jadhav et al. (2012) study developed and assessed an approach to perform miRNA overexpression and screen their effect on bioindustrially relevant phenotypes, such as growth, viability, and productivity. The average growth rate shows an increase of 15.4% for miR-17 and 7.2% for miR-221, respectively. For miR-21 and miR-210, no changes in growth rates were observed. Moreover, in the cases of miR-21 and miR-210, the authors observed a decrease in productivity. They suggested that the logical next step is designing an appropriate knockdown approach, such as a sponge, to reduce the intracellular level of these miRs in the hope of increasing the product yield (Jadhav et al. 2012). Maccani et al. (2014) observed a general increase of some miRNA expression levels in the producing cell lines, which indicates that miRNAs play an essential role in the regulation of processes involved or caused by recombinant protein synthesis and secretion. Nevertheless, these authors observed downregulation of miR-21-5p in recombinant cell lines. They mentioned that miR-21-5p overexpression reduces specific productivity. Consequently, their study suggested that a knockdown of miR-21-5p could increase particular productivity. The authors claim that all miRNAs they described, including miR-21, have been associated with cell growth and/or apoptosis, so their relation to productivity may only be indirect (Maccani et al. 2014). In the recent study by Belliveau et al. (2024), miR-21, known for its anti-apoptotic role in other mammalian cell lines, was investigated in CHO cells. When miR-21 was overexpressed, it negatively impacted the growth and viability of CHO cells during the late exponential-phase culture. This result was unexpected, given the reported anti-apoptotic role of miR-21 in other mammalian cell lines (Belliveau et al. 2024). Unlike human cancer cells that overexpress miR-21, CHO cells do not exhibit an increase in growth rate upon miR-21 expression. On the other hand, upon applying CM21D against miR-21, we observed an increased growth rate of 1.3-fold compared to the CM21SD-expressing clonal cell. The productivity of the cell pool co-expressing hrsACE2 and CM21D with hrsACE2 expressing clonal cells were compared using ELISA. We observed a twofold increase in hrsACE2 productivity and a 1.1-fold increase in growth rate compared to hrsACE2 expressing clonal cells. We can, therefore, conclude that sponging-induced knockdown of miR-21 could result in an increase in growth rate, which may be associated with enhanced productivity in CHO cells. We recommend additional investigations into the possible molecular pathways that underlie cell growth and productivity improvement following the inhibition of miR-21 expression.

## Supplementary Information

Below is the link to the electronic supplementary material.

Supplementary file1 (PDF 1540 KB)

## Data Availability

All data generated or analyzed during this study are included in this published paper and its supplementary information file.
