# Supplementary material for: CHO cell engineering via targeted integration of circular miR-21 decoy using CRISPR/RMCE hybrid system
Source: Appl Microbiol Biotechnol. 2024 Aug 9;108:434. doi: 10.1007/s00253-024-13266-4 (PMC11315787; doi:10.1007/s00253-024-13266-4)
Supplement: Supplementary file 1 [file 253_2024_13266_MOESM1_ESM.pdf]

Supplemental Material

Applied Microbiology and Biotechnology

CHO Cell Engineering via Targeted Integration of Circular miR-21 Decoy Using CRIS-PITCh/Bxb1 Hybrid System

Setare Adibzadeh[1,2], Shahin Amiri[1,2], Farzaneh Barkhordari[1], Seyed Javad Mowla[3], Hadi Bayat[4], Samaneh Ghanbari[1], Faezeh Faghihi[*5], Fatemeh Davami[1*]

[1]Department of Medical Biotechnology, Biotechnology Research Center, Pasteur Institute of Iran, Tehran, Iran

[2]Student Research Committee, Pasteur Institute of Iran, Tehran, Iran

[3]Department of Molecular Genetics, Faculty of Biological Sciences, Tarbiat Modares University, Tehran, Iran

[4] Biochemical Neuroendocrinology, Institut de Recherches Cliniques de Montréal (IRCM), affiliated to the Division of Experimental Medicine, Faculty of Medicine and Health Sciences, McGill University, Montréal H2W 1R7, Canada.

[5]Cellular and Molecular Research Center, Iran University of Medical Sciences, Tehran, Iran

***Correspondence:**

Fatemeh Davami, PharmD/PhD, Full Professor at Pasteur Institute of Iran, Tehran, Iran. Email: f.davami@gmail.com Tel: +982166402443 Fax: +982166465132

***Co-correspondence:**

Faezeh Faghihi, Ph.D., Associate Professor at Iran University of Medical Sciences, Email: faezefaghihi@yahoo.com Tel: +982144604800 Fax: +982188622578

Table S1 Sequence of primers and stem loops.

No	Primer name	Function	5' to 3' sequence
1	5′junction F	5' junction amplification	GGACAGCACCCAGACTTACACC
2	5' junction R	5' junction amplification	CCTACCCGCTTCCATTGCTCAG
3	3' junction F	3' junction amplification	GCAACCTCCCCTTCTACGAGC
4	3' junction R	3' junction amplification	CATCGGGTAGATGAGCCCAC
5	Genomic F	Out-out amplification	GTCCAGGCTCCCATTTCTGACA C
6	Genomic R	Out-out amplification	CTTCTGCCTTCTGGTGGTGGAG
7	Sponge-Convergent F	Linear and circular structure of sponge amplification and qRT-PCR	AGTGCGGCCACCCTCAAGATC
8	Sponge-Convergent R	Linear and circular structure of sponge amplification and qRT-PCR	TTCAACACCGCGGCCACTTATG
9	Sponge-Divergent F	Circular structure of sponge amplification	CAGAACATAAGCTAGGCGTAT GTGG
10	Sponge-Divergent R	Circular structure of sponge amplification	GTTCTGATGTTGATCTTGAGGG TGGC
11	Scramble-Convergent F	Linear and circular structure of scramble amplification and qRT-PCR	GTGCGGCCACCCTCAAGATAA
12	Scramble-Convergent R	Linear and circular structure of scramble amplification and qRT-PCR	TTCAACACCGCGGCCACTTATG

13	Scramble-Divergent F	Circular structure of scramble amplification	ACCACTAAATAACATGGGCCCACAT
14	Scramble-Divergent R	Circular structure of scramble amplification	TAGTGGTCGCATTATCTTGAGGGTGG
15	*TK*135 F	Amplification of *TK* for qRT-PCR	AGCAGAAAATGCCCACGCTA
16	*TK*135 R	Amplification of *TK* for qRT-PCR	AGTAAGTCATCGGCTCGGGT
17	*GFP* F	Amplification of *EGFP* for qRT-PCR	CAGAAGAACGGCATCAAGGT
18	*GFP* R	Amplification of *EGFP* for qRT-PCR	GTGCTCAGGTAGTGGTTGTC
19	*ACTB* F	Amplification of *ACTB* for qRT-PCR	GTCCTACCTGCCTTGACTACTT
20	*ACTB* R	Amplification of *ACTB* for qRT-PCR	TACGACCAGAGGCATACAGGG
21	miR-21 stem-loop	Synthesis of miR-21 cDNA	GTCGTATGCAGAGCAGGGTCCGAGGTATTCGCACTGGATACGACTCAACA
22	miR-21 F	Amplification of miR-21 for qRT-PCR	CCGGCCTAGCTTATCAGACTG
۲3	U6 stem-loop	Synthesis of U6 snoRNA cDNA	GTCGTATGCAGAGCAGGGTCCGAGGTATTCGCACTGGATACGACCGCTTC
۲4	U6 F	Amplification of U6 snoRNA for qRT-PCR	CTCGCTTCGGCAGCAC
۲5	Universal R	Amplification of miR-21 and U6 snoRNA for qRT-PCR	GTGCAGGGTCCGAGGT
۲6	*Trim33* F	Amplification of *Trim33* for qRT-PCR	TCTACCCCCACAGCCTACAAG

27	*Trim33* R	Amplification of *Trim33* for qRT-PCR	CGACTGCCAGTGCTAGATGTAC
28	*Pdcd4* F	Amplification of *Pdcd4* for qRT-PCR	GGTGGACGTGAAAGATCCCAA C
29	*Pdcd4* R	Amplification of *Pdcd4* for qRT-PCR	CCTCATTTGTGTCTCCGTGCTC
۳0	*Atp11b* F	Amplification of *Atp11b* for qRT-PCR	CTCTTGCACTCAGGGAGCAT
31	*Atp11b* R	Amplification of *Atp11b* for qRT-PCR	CCAACAGCCAATGTGATCGGT

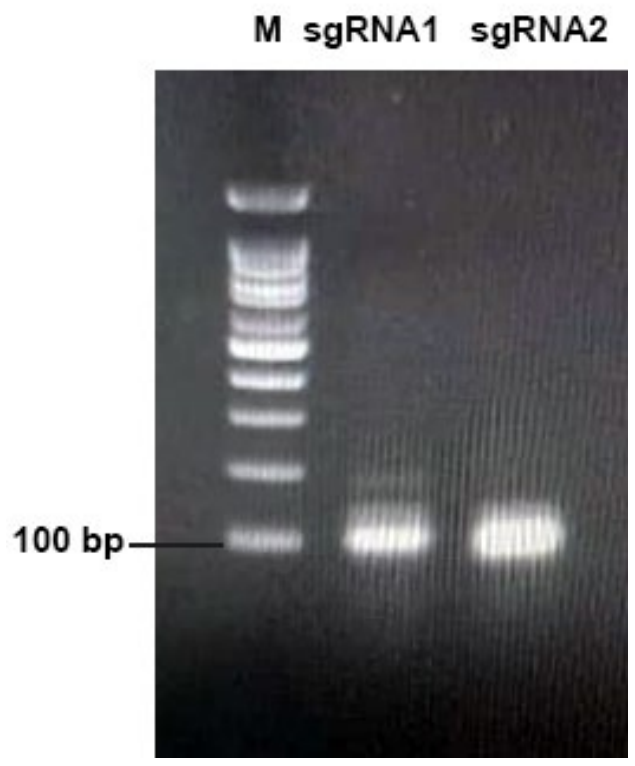

Fig. S1 2% agarose gel electrophoresis of the in vitro transcription product. sgRNA1 corresponds to the PITCh sgRNA, while sgRNA2 represents the genome-targeting sgRNA. The expected size for both sgRNAs is 100 bp. The M lane contains the 100 bp DNA marker.

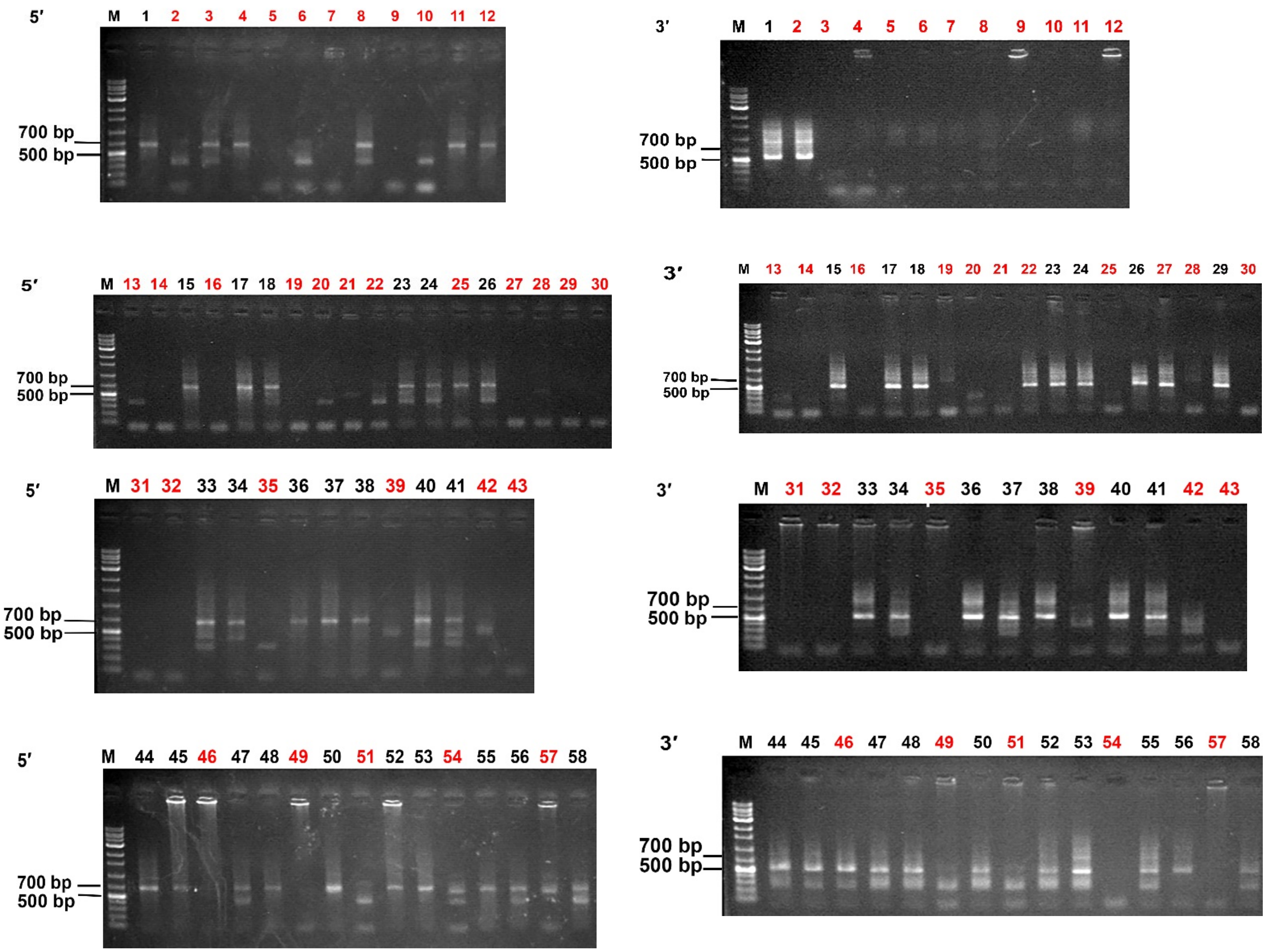

Fig. S2 The results of agarose gel electrophoresis for the 5′/3′ junction PCR of the landing pad on RNP based delivery system single clones. The expected PCR product sizes were 666 bp for the 5′ junction and 622 bp for the 3′ junction PCRs. Positive clones in both the 5′ and 3′ junction PCRs are indicated by black numbers and negative clones for either 5′ or 3′ junction PCRs are indicated by red numbers. The M lane corresponds to the 1 kb DNA marker.

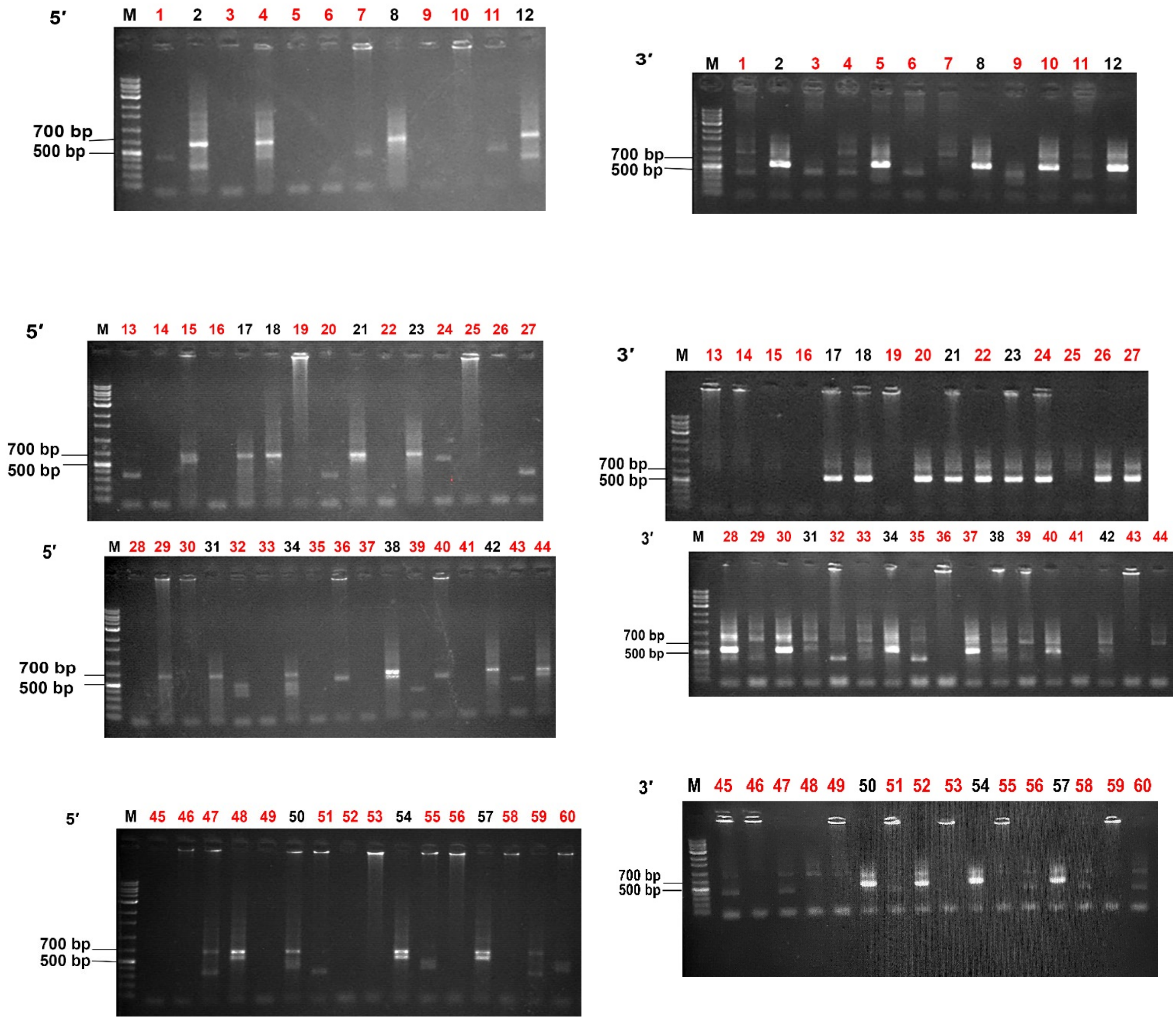

Fig. S3 The results of agarose gel electrophoresis for the 5′/3′ junction PCR of the landing pad on plasmid-based delivery system single clones. The expected PCR product sizes were 666 bp for the 5′ junction and 622 bp for the 3′ junction PCRs. Positive clones in both the 5′ and 3′ junction PCRs are indicated by black numbers and negative clones for either the 5′ or 3′ junction PCRs are indicated by red numbers. The M lane corresponds to the 1 kb DNA marker.

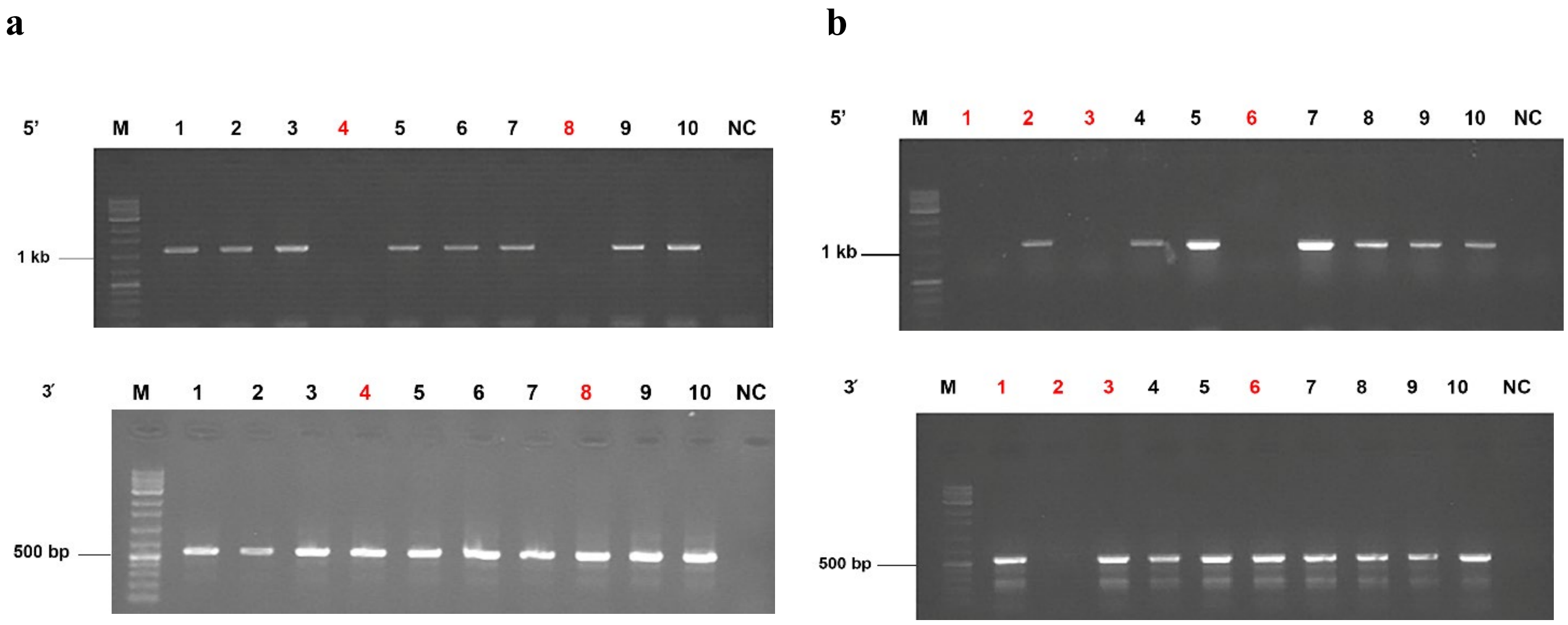

Fig. S4 The results of agarose gel electrophoresis for the 5′/3′ junction PCR on retargeted single clones. a Agarose gel electrophoresis for the 5′/3′ junction PCR on CM21D expressing single clones. **b** Agarose gel electrophoresis for the 5′/3′ junction PCR on CM21SD expressing single clones. The expected PCR product sizes were approximately 1360 bp for the 5′ junction and 646 bp for the 3′ junction PCR. Positive clones in both the 5′ and 3′ junction PCRs are indicated by black numbers and negative clones for either the 5′ or 3′ junction PCRs are indicated by red numbers. The M lane corresponds to the 1 kb DNA marker, while the NC lane represents the negative control.

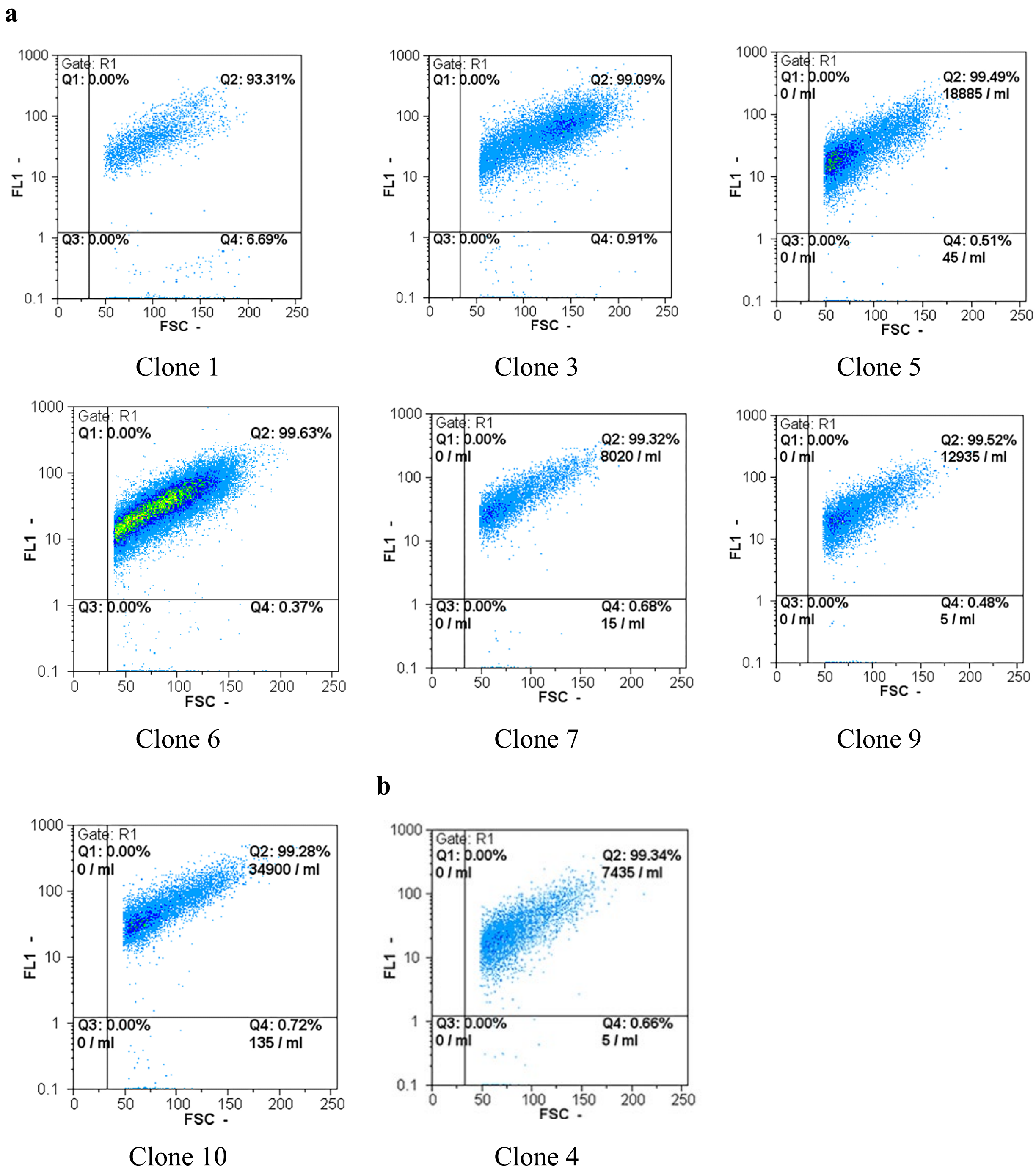

Fig. S5 EGFP expression analysis in retargeted single clones by flow cytometry. a Single clones retargeted with CM21D and expressed EGFP > 99% were selected for the next step. **b** A single clone retargeted with CM21SD, which also exhibited EGFP > 99%, was chosen next step.

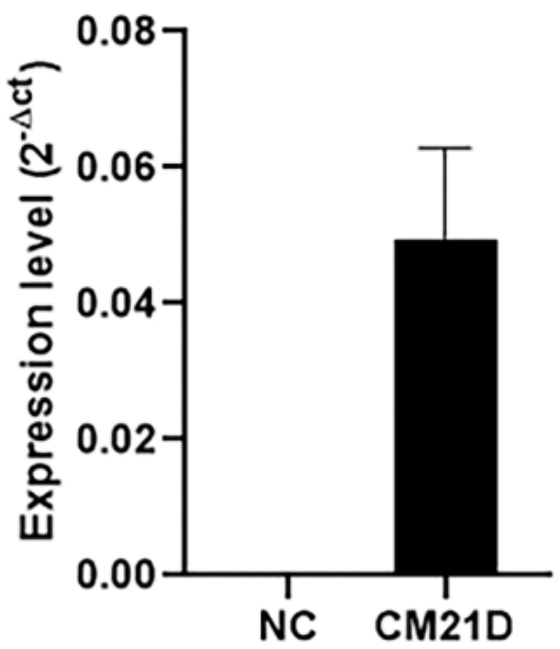

Fig. S6 Validating the expression of CM21D. Expression level of CM21D and CM21SD was measured using qRT-PCR (based on the formula $2^{(-\Delta Ct)}$). The error bars correspond to the standard deviations observed in technical replicates (n = 3).

a

Tube	Concentration (ng/μl)	Absorbance
1	20	1.004
2	10	0.69
3	5	0.4395
4	2.5	0.303
5	1.25	0.1975
6	0.625	0.1385
7	0.3125	0.1115

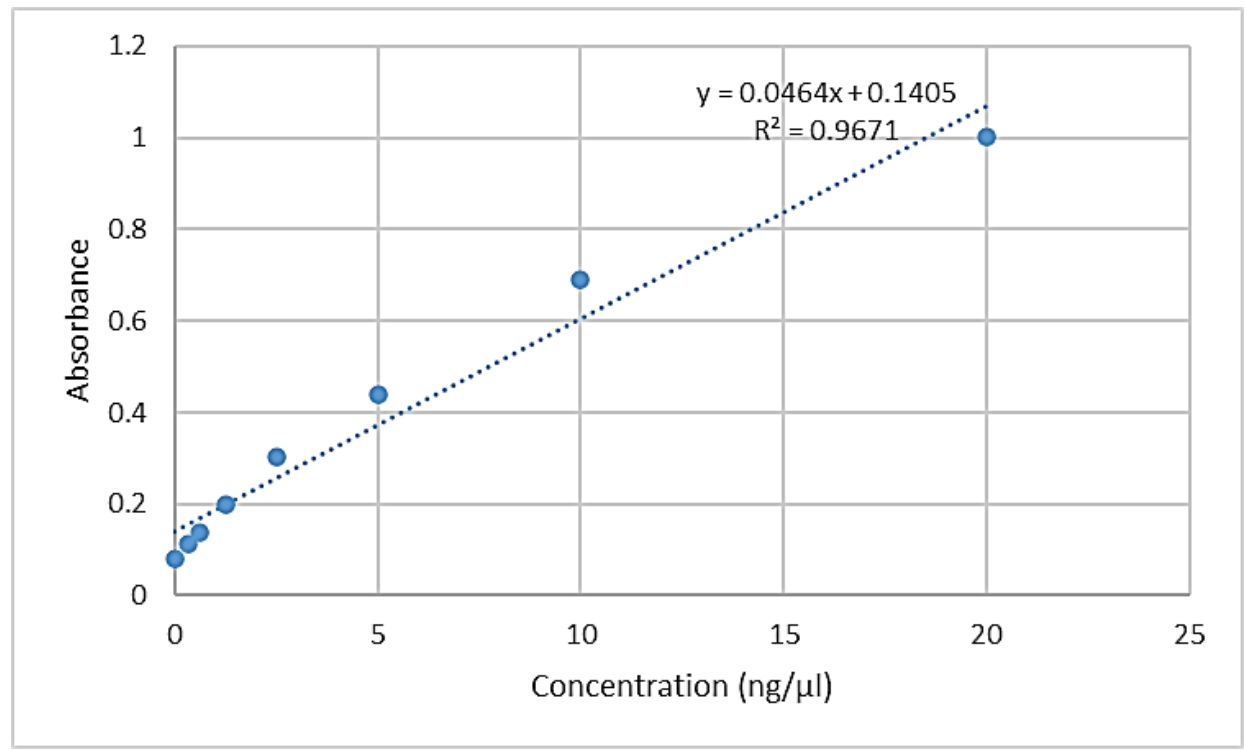

b

Test group

OD	Concentration (μg/ml)
0.721	6.143 μg/ml
0.648	8.042 μg/ml
0.5445	8.507 μg/ml

Control group

OD	Concentration (μg/ml)
0.452	3.263μg/ml
0.362	3.442μg/ml
0.3493	4.326μg/ml

Fig. S7 Determination of hrsACE2 productivity using ELISA assay. a The standard curve is graphed with optical density (OD) plotted against the logarithm of protein concentration, as indicated in the table. **b** The concentration of hrsACE2 was determined in both the test and control groups. Three different dilutions were used: 1/500, 1/750, and 1/1000. Each dilution was tested in triplicate.
